# Epigallocatechin Gallate Attenuates CaOx Crystal-Induced Renal Tubular Injury to Inhibit CaOx Nephrolithiasis via GRP94/PI3K/AKT Signaling

**DOI:** 10.34133/bmr.0271

**Published:** 2025-11-17

**Authors:** Jian Wu, Minghui Liu, Meng Gao, Yongchao Li, Youjie Zhang, Liang Tang, Hao Yu, Zhangcheng Liao, Yu Cui, Feng Zeng, Hequn Chen, Zewu Zhu

**Affiliations:** ^1^ Department of Urology, Xiangya Hospital, Central South University, Changsha, Hunan, China.; ^2^ National Clinical Research Center for Geriatric Disorders, Xiangya Hospital, Central South University, Changsha, Hunan, China.; ^3^ Department of Urology, General Hospital of Ningxia Medical University, Yinchuan, Ningxia Hui Autonomous Region, China.; ^4^ Institute of Physiology, University of Zurich, Zurich, Switzerland.; ^5^ Department of Urology, Peking Union Medical College Hospital, Chinese Academy of Medical Science and Peking Union Medical College, Beijing, China.; ^6^ Department of Internal Medicine, Section Endocrinology, Yale University School of Medicine, New Haven, CT, USA.

## Abstract

Although tea consumption has been suggested to affect kidney stone formation, epidemiological evidence remains inconsistent, and the underlying molecular mechanisms are unclear. To assess the association between tea intake and kidney stone risk, we initially conducted a prospective cohort analysis of 481,393 participants from the UK Biobank and a 2-sample Mendelian randomization (MR) analysis. Our findings revealed that heavy tea drinkers (>5 cups/day) had a significantly reduced risk of kidney stones (hazard ratio: 0.79, 95% confidence interval [CI]: 0.72 to 0.86, *P* < 0.001), and MR analyses confirmed a causal association (inverse variance weighted OR: 0.45, 95% CI: 0.32 to 0.62, *P* < 0.001). We next explored the effect of epigallocatechin gallate (EGCG), the main bioactive component in tea, on calcium oxalate (CaOx) stone formation. EGCG was found to inhibit the glucose-regulated protein 94/phosphatidylinositol 3-kinase/protein kinase B (GRP94/PI3K/AKT) pathway in human proximal renal tubular epithelial cells, thereby attenuating CaOx crystal-induced oxidative stress and inflammation, and inhibiting crystal-cell adhesion. This finding aligned with the observation that the activated GRP94/PI3K/AKT pathway was positively associated with inflammation-related molecules in renal papillary tissues of CaOx stone formers. Moreover, to enhance renal targeting and therapeutic potential, we synthesized cell membrane-coated EGCG-loaded poly(lactic-co-glycolic acid) (TP-EGCG) nanoparticles, which enhanced renal EGCG delivery and substantially reduced CaOx crystal deposition in a mouse model of CaOx nephrolithiasis. In conclusion, tea consumption protects against kidney stone formation, an effect exerted by EGCG through the GRP94/PI3K/AKT axis, and our novel TP-EGCG nanoparticles show strong potential for targeted prevention and treatment.

## Introduction

Kidney stones represent a common chronic disease worldwide, posing a notable public health challenge due to their high prevalence and recurrence rates. Globally, the prevalence of kidney stones varies across regions and populations, affecting approximately 1% to 13% of individuals [1]. Despite advancements in minimally invasive urological surgery, kidney stones exhibit notably high recurrence rates, approaching 50% within 5 to 10 years and 75% within 20 years [2], underscoring an urgent clinical need for effective preventive strategies.

Tea, one of the most widely consumed beverages globally, has long been speculated to influence kidney stone formation. However, observational studies examining the association between tea consumption and kidney stones have yielded conflicting findings [3,4]. While the protective effects of tea may be attributed to its rich polyphenol content with strong antioxidant properties [5], its high oxalate concentration could have a potentially lithogenic effect [3]. Crucially, prospective studies systematically addressing this relationship remain limited. The UK Biobank (UKB), a prospective cohort enrolling over 500,000 UK participants, provides an exceptional resource for addressing these limitations [6]. Additionally, Mendelian randomization (MR), utilizing genetic variants as instrumental variables (IVs), offers a robust approach to clarify causal relationships while minimizing confounding and reverse causation biases [7]. Thus, integrating MR analysis with prospective cohort data from UKB could yield strong evidence regarding the causal relationship between tea consumption and kidney stone formation.

Calcium oxalate (CaOx) stones are the most prevalent subtype of kidney stones, accounting for about 80% [8]. Currently, the molecular mechanisms underlying CaOx stone formation are not completely understood. Accumulating evidence indicates that CaOx stone formation is a multifactorial pathological biomineralization process involving complex interactions between physicochemical events and biological responses [9,10]. Urine supersaturation serves as the primary driving force behind kidney stone formation [9]. Under the conditions of supersaturation, CaOx crystals are generated, leading to oxidative stress and inflammatory responses that subsequently cause renal tubular cell injury [9,10]. These oxidative and inflammatory events play a crucial role in promoting crystal adhesion [11]. Moreover, Randall’s plaque, characterized by calcium phosphate deposits within the renal papillary interstitium, provide crucial sites for CaOx crystal attachment, thereby facilitating the CaOx crystal retention, aggregation, and eventual stone formation [12].

Epigallocatechin gallate (EGCG), a primary bioactive polyphenol in green tea, exhibits potent antioxidant and anti-inflammatory properties [13], implying potential therapeutic benefits against CaOx stones. Notably, flavonoids as a broader class of polyphenolic compounds have been increasingly recognized for their nephroprotective properties. For instance, quercetin has shown reno-protective effects in animal models of kidney injury through the attenuation of oxidative stress and inflammation [14]. These findings suggest that EGCG may act through similar mechanisms, reinforcing the potential of dietary flavonoids in kidney stone prevention and treatment. In vitro studies have demonstrated that EGCG mitigated cellular damage induced by CaOx crystals [15–17]; nevertheless, the exact underlying mechanism remains unclear. Additionally, the clinical application of EGCG is constrained by its low bioavailability and poor chemical stability [18]. Therefore, enhancing EGCG bioavailability and targeted delivery remains a critical challenge. Nanoparticle-based drug delivery systems present a promising approach to overcome these limitations. Poly(lactic-co-glycolic acid) (PLGA) nanoparticles, characterized by excellent biodegradability and controlled-release capabilities, have been successfully utilized to enhance EGCG’s therapeutic efficacy in cancer treatment [19,20]. Furthermore, cell membrane-coating technologies can markedly improve nanoparticle targeting efficiency and extend systemic circulation [21]. While such technologies have primarily been explored in the field in cancer [22], their potential for kidney stone prevention and treatment remains largely unexplored. Consequently, we developed cell membrane-coated EGCG-loaded PLGA nanoparticles to investigate their therapeutic potential in vivo, aiming to achieve renal-specific delivery and enhanced efficacy against CaOx stones.

Overall, this study systematically evaluates the complex relationship between tea consumption and kidney stones by integrating MR analysis and prospective cohort study. Concurrently, we investigate the protective mechanisms of EGCG against CaOx crystal-induced cellular injury in vitro and further validate the therapeutic efficacy of our novel nanoparticle delivery system in vivo. Our findings aim to provide robust scientific evidence to support clinical translation for EGCG and broaden the application of nanotechnology in urological therapeutics.

## Materials and Methods

### Cohort study

The cohort study data were obtained from the UKB, a large-scale, population-based cohort that recruited 502,505 community participants aged 37 to 73 years from 22 assessment centers across England, Scotland, and Wales between 2006 and 2010 [6]. At baseline, participants underwent physical examinations and completed electronic questionnaires to collect demographic, lifestyle, and health-related information. Additionally, all participants provided informed consent for long-term health follow-up, including linkage to electronic medical records. The study protocol was approved by the National Health Service (NHS) and the North West Multi-Center Research Ethics Committee (Ethics Approval Number: 11/NW/0382).

**Fig. 1.**
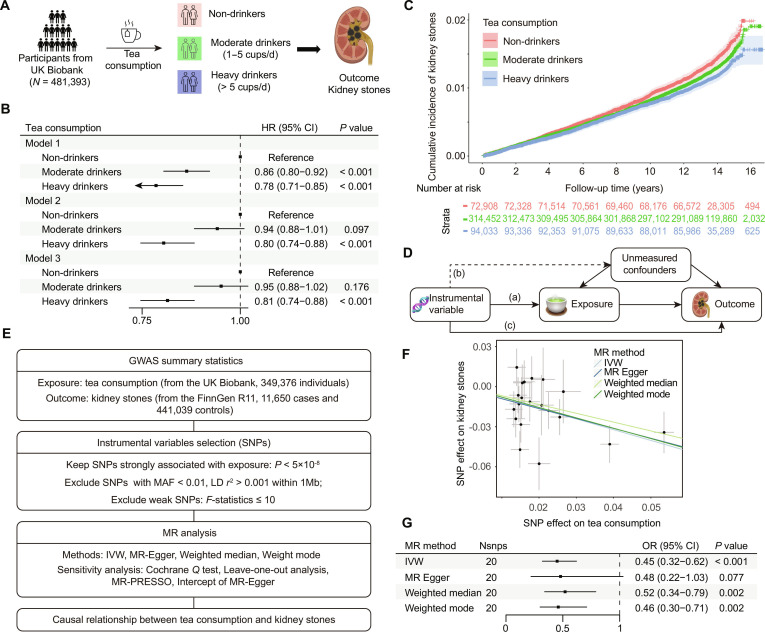
Association between tea consumption and the risk of kidney stone. (A) Design and grouping of the prospective cohort study based on the UK Biobank. (B) Cox proportional hazards models evaluating the association between tea consumption and kidney stone risk. Model 1, adjusted for sex, age, and ethnicity; Model 2, adjusted for educational attainment, average total household income, smoking, drinking, BMI, physical activity, and diet based on Model 1; Model 3, adjusted for hypertension, T2DM, CVD, use of medication including insulin, antihypertensive drugs, and antilipemic agent based on Model 2. (C) Cumulative incidence of kidney stones. (D) Three basic assumptions of MR analysis: (a) reliable association; (b) no association; and (c) no independent association. (E) Design of MR analysis. (F) Scatter plot of the causal relationship between tea consumption and kidney stones using 4 MR methods. (G) Association of tea consumption with kidney stones in MR analysis.

For this analysis, individuals with a prior diagnosis of kidney stones at baseline (*N* = 3,232) and those with missing tea consumption data (*N* = 17,526) were excluded, resulting in a final sample of 481,393 participants (Fig. 1A). Tea consumption was assessed through a self-reported questionnaire: “How many cups of tea (including black and green tea) do you drink per day?”, categorizing participants into non-tea drinkers (0 cups/day), moderate tea drinkers (1 to 5 cups/day), and heavy tea drinkers (>5 cups/day). Kidney stones were identified using International Classification of Diseases, 10th Revision codes (N20/N23) from linked medical records. Covariates included demographic characteristics (age, sex, ethnicity, and education level), socioeconomic status (Townsend Deprivation Index), lifestyle factors (smoking status, alcohol consumption, and physical activity), body mass index, dietary quality (Healthy Diet Score), baseline comorbidities (diabetes, hypertension, and heart disease), and medication usage (insulin, blood pressure medication, and cholesterol-lowering medication). Detailed descriptions of the assessment methods of these covariates are consistent with our previous research [23].

### Two-sample MR analysis

This study utilized a 2-sample MR approach to explore the causal relationship between tea consumption and kidney stones, using publicly available genome-wide association study (GWAS) data. Genetic IVs for tea consumption were identified from the UKB GWAS dataset (Neale Lab, *N* = 349,376), while kidney stone data were sourced from the FinnGen Consortium (Release 11; 11,650 cases and 441,039 controls). Detailed selection of IVs and MR assumptions are presented in the Supplementary Materials.

### Cell culture

Human proximal renal tubular epithelial cell line (HK-2, RRID: CVCL_0302) and mouse kidney epithelial cells line (TCMK-1, RRID: CVCL_2772) were obtained from the Central South University Cell Bank and Wuhan Procell Company (China), respectively. Cells were cultured in Minimum Essential Medium (meilunbio, China) supplemented with 10% fetal bovine serum (meilunbio, China) and 1% penicillin–streptomycin (Abiowell, China), at 37 °C in a humidified atmosphere with 5% CO₂. A CaOx crystal-induced cell injury model was established following the protocol described in reference [24]. HK-2 cells were treated with 100 μg/ml monohydrate CaOx (COM) crystals for 24 h. EGCG (meilunbio, China) was dissolved in dimethyl sulfoxide (DMSO; Abiowell, China) to prepare a 40 mM stock solution, which was then serially diluted in complete medium to obtain final concentrations ranging from 5 to 160 μM (5, 10, 20, 40, 80, and 160 μM) for experimental treatments.

### Clinical sample collection and processing

Renal papillary tissue specimens for this study were approved by the Ethics Committee of Xiangya Hospital, Central South University (Approval No. 202103089). Randall’s plaque (RP) tissues were obtained from patients with CaOx stones who underwent percutaneous nephrolithotomy; normal renal papillae (NRP) tissues were collected from patients undergoing radical nephrectomy, from nonstone and noncalcified regions at least 2 cm from the tumor margin, as described in our previous study [25]. Baseline clinical characteristics of included patients are provided in Table S1.

### Cell viability assay, lactate dehydrogenase release assay, and Calcein/PI staining assay

Cell viability and death were assessed using the Cell Counting Kit-8 (CCK-8) assay (meilunbio, China) and Calcein/PI dual staining kit (Beyotime, China). Cell damage was assessed using the lactate dehydrogenase (LDH) release assay (Elabscience, China), as described in the Supplementary Materials.

### Reactive oxygen species detection assay

Reactive oxygen species (ROS) levels were measured using the DCFH-DA assay (meilunbio, China) and dihydroethidium (DHE; Beyotime, China) staining. Intracellular ROS levels were assessed using the fluorescent probe DCFH-DA. After experimental treatments, the DCFH-DA probe was diluted 1:1,000 in serum-free medium and applied to the cells. The samples were incubated under light-protected conditions at 37 °C for 30 min. ROS levels were then analyzed using either fluorescence microscopy or flow cytometry. For fluorescence imaging, Hoechst 33342 nuclear staining (Abiowell, China) was used for cell localization. For flow cytometry analysis, cells were trypsinized posttreatment to obtain a single-cell suspension, then incubated with the DCFH-DA probe. For tissue-level assessment, frozen kidney sections were subjected to DHE staining to detect the ROS levels. Detailed procedures for DHE staining are provided in THE Supplementary Materials.

### Measurement of oxidative stress markers

This study assessed 4 oxidative stress markers: malondialdehyde (MDA), hydrogen peroxide (H_2_O_2_), total superoxide dismutase (T-SOD), and total glutathione (T-GSH). All assays were conducted according to the manufacturer’s instructions for reagent kits from Elabscience (China). The corresponding kit catalog numbers were E-BC-K028-M, E-BC-K102-M, E-BC-K020-M, and E-BC-K097-M.

### Crystal adhesion assay

The adhesion of COM crystals to the surface of HK-2 cells was assessed using fluorescence imaging. HK-2 cells were seeded into 6-well plates and cultured until reaching the desired confluence, followed by experimental treatments. After treatment, nonadherent CaOx crystals were removed by washing the cells 3 times with phosphate-buffered saline (PBS) (5 min per cycle). Cells were then fixed with 5% paraformaldehyde at room temperature for 15 min, followed by PBS rinsing. A total of 500 μl of ready-to-use 4',6-diamidino-2-phenylindole (DAPI) staining solution was added to each well, and the plate was incubated in the dark for 5 min. Excess dye was removed by 3 additional PBS washes. Images were acquired using an inverted fluorescence microscope (Lecia, Germany), including bright-field images to observe crystal adhesion morphology and fluorescence images to visualize DAPI-stained nuclei.

### Immunofluorescence staining

Cells were rinsed with PBS and fixed with 4% paraformaldehyde at room temperature for 10 to 15 min. To minimize nonspecific binding, cells were blocked with 5% goat serum (Solarbio, China) at room temperature for 30 min. Cells were then incubated with the primary antibody, diluted according to the manufacturer’s instructions, overnight at 4 °C. The next day, the primary antibody solution was removed, and cells were washed with PBS before incubation with a fluorescently labeled secondary antibody (1:400 dilution) for 1 h in the dark. The nuclei were stained with DAPI staining solution (Abiowell, China) and incubated in the dark for 5 min. After extensive PBS washing, the slides were mounted with a coverslip. Multichannel fluorescence imaging was performed using an inverted fluorescence microscope. The detailed information about the primary and secondary antibody is provided in Table S2.

### Quantitative real-time reverse transcription PCR

Total RNA was extracted using the SteadyPure Rapid RNA Extraction Kit (Accurate Biology, China) according to the manufacturer’s instructions. The purity and concentration of the extracted RNA were assessed using a NanoDrop spectrophotometer (Thermo Fisher, USA). Complementary DNA was synthesized by reverse transcription using the Evo M-MLV reverse transcription premix kit (Accurate Biology, China). qPCR was performed using the SYBR Green Pro Taq HS Premix qPCR Kit II (with ROX) (Accurate Biology, China) on a real-time fluorescence qPCR instrument (Eppendorf, Germany). Thermal cycling conditions were set according to the kit protocol. GAPDH was used as the internal control gene, and target gene expression levels were quantified using the 2^−ΔΔCT^ method. The primer sequences are provided in Table S3.

### Western blot

Western blot (WB) was performed as described in our previous study. Briefly, proteins were separated by 10%–12% gradient SDS-PAGE (sodium dodecyl sulfate polyacrylamide gel electrophoresis) and subsequently transferred onto a polyvinylidene fluoride (PVDF) membrane (Millipore, USA). The membrane was blocked with rapid blocking buffer (Epizyme, China) at room temperature for 15 min. Next, the membrane was incubated overnight at 4 °C with a specific primary antibody, followed by a 1-h incubation with a horseradish peroxidase (HRP)-conjugated secondary antibody (1:5,000 dilution) at room temperature. After incubation with ECL chemiluminescent substrate (Zenbio, China), protein bands were visualized using a chemiluminescence imaging system (Amersham, UK), and band intensities were quantified using ImageJ software.

### Calcium ion quantification assay

After treatment, cells were rinsed with PBS to remove unbound crystals. Then, 1 ml of 0.1 mM HCl solution was added to each well, and the plates were incubated overnight at 4 °C to dissolve the crystals and release calcium ions. Following lysis, the samples were centrifuged, and the supernatant was collected and diluted 1:2 with deionized water. Calcium ion levels were measured using a calcium colorimetric assay kit (Elabscience, China). Simultaneously, total cellular protein was extracted, and its concentration was determined using the bicinchoninic acid (BCA) assay (Epizyme, China). Calcium ion levels were normalized to total protein content for data analysis.

### RNA sequencing and differential expression analysis

HK-2 cells were treated with COM alone or COM combined with EGCG for 24 h (*n* = 3 per group). Total RNA was extracted from each T25 culture flask using 1 ml of Trizol reagent (Accurate Biology, China). The extracted RNA samples were flash-frozen in liquid nitrogen and sent to Biomarker Technologies Co., Ltd (China) for paired-end sequencing using the Illumina HiSeq X Ten platform. Differentially expressed genes (DEGs) were identified using the limma package, with the screening criteria set as an false discovery rate-adjusted *q* value < 0.05 and |log₂FC| > 1. Results were visualized using a volcano plot. Kyoto Encyclopedia of Genes and Genomes (KEGG) pathway enrichment analysis was performed using the clusterProfiler package, and the results were visualized as bar plots. The raw sequencing data and list of DEGs are available in Table S4.

### Cell transfection

The HSP90B1 overexpression vector (pCMV3-HSP90B1; Sino Biological, China) was amplified in competent cells and purified using an endotoxin-free plasmid extraction kit. HK-2 cells were transfected with the purified plasmid using Lipofectamine 3000 (Thermo Fisher, USA) for gene overexpression. For gene knockdown experiments, a specific HSP90B1 siRNA designed by RiboBio (China) was used, with si-Ctrl serving as a negative control. Transfection was carried out using Lipofectamine 3000. Transfection efficiency was validated by quantitative real-time reverse transcription PCR (qRT-PCR) for mRNA levels and WB for protein expression. Details of the plasmid vectors and siRNA sequences are listed in Table S5.

### H&E, Von Kossa, and immunohistochemistry staining

Paraffin sections were baked at 60 °C, dewaxed with xylene, and rehydrated through a graded ethanol series. Calcium salt deposition in tissues was detected using the Von Kossa staining kit (Solarbio, China), and hematoxylin and eosin (H&E) staining was performed with the H&E kit (Solarbio, China). For immunohistochemistry (IHC) staining, antigen retrieval was performed using microwave heating, followed by washing with TBST (Tris-buffered saline with Tween-20). Sections were blocked with 5% goat serum for 1 h at room temperature, then incubated sequentially with a primary antibody (overnight at 4 °C) and an HRP-conjugated secondary antibody (1 h at room temperature). After 3,3′-diaminobenzidine staining, nuclei were counterstained with hematoxylin, followed by differentiation and bluing to adjust contrast. Microscopic images were acquired and analyzed semiquantitatively using ImageJ, as reported by our previous study [26].

### Molecular docking

The 3-dimensional molecular structure of the small-molecule compound EGCG (PubChem CID: 65064) was obtained from the PubChem database. The crystal structure of the protein receptor was retrieved from the Protein Data Bank (PDB). The PDB IDs for 3 protein receptors were 4NH9 for GRP94, 4H6J for HIF1A, and 5J2X for HSP90-α. Molecular docking was performed using the CB-DOCK2 online platform (https://cadd.labshare.cn/cb-dock2/), which is based on the AutoDock Vina algorithm and integrates protein pocket recognition and docking conformation prediction functions [27]. Default parameters were used during the docking process.

### Cellular thermal shift assay

Total protein from HK-2 cells was extracted by repeated freeze-thawing. After washing with precooled PBS, the cells were scraped and subjected to 3 cycles of freeze-thawing in liquid nitrogen for lysis. The lysates were centrifuged at 12,000 rpm for 15 min at 4 °C to collect the supernatant. The extracted total protein was divided into an EGCG treatment group (final concentration of 40 μM) and a DMSO control group. The samples were incubated at room temperature for 2 h and then aliquoted into PCR tubes. Thermal denaturation was performed in a PCR machine with a temperature gradient from 37 to 62 °C (3 min at each temperature). The reaction was stopped by placing the samples on ice. After centrifugation, the supernatant was collected and mixed with protein loading buffer (4:1), then denatured in a metal bath at 95 °C for 10 min. Thermal stability changes of the target protein were finally assessed by WB.

### Preparation and characterization of nanoparticles

PLGA nanoparticles were prepared using an ultrasonic emulsification method. PLGA (50 mg; Sigma, USA) was first dissolved in dichloromethane (Aladdin, China) and loaded with either EGCG (5 mg, in DMSO) or DIR (protected from light; Sigma, USA). The solution was slowly added dropwise to a precooled aqueous phase of PVA (50 mg/50 ml; Sigma, USA) to form a nanoemulsion. After 10 h of magnetic stirring to remove the organic solvent, the solution was centrifuged and washed to obtain EGCG-PLGA-NPs or DIR-PLGA-NPs (concentration: 100 μg/ml). The control group contained only DMSO.

TCMK-1 cell membranes were isolated by trypsin digestion, differential centrifugation, and ultrasonic disruption to extract membrane proteins (quantified using the BCA assay). The extracted membrane proteins were mixed with nanoparticles at a membrane protein-to-nanoparticle ratio of 200 μg:1 mg and ultrasonically treated on ice for 15 min to enable physical coating. Unbound membrane components were removed by centrifugation to obtain membrane-coated nanoparticles (TP-EGCG/DIR). Nanoparticle morphology and distribution were examined using transmission electron microscopy (TEM) (Hitachi, Japan).

### In vivo targeting and retention evaluation of membrane-coated nanoparticles

Mice were injected via the tail vein with TCMK-1 cell membrane-coated DIR-PLGA nanoparticles (TP-DIR NPs, 10 mg/kg) or noncoated control nanoparticles (P-DIR NPs). Fluorescence signals at 780 nm were dynamically monitored using an IVIS live imaging system (Thermo Fisher, USA) at 4, 8, 16, 24, 32, and 48 h postinjection. At 48 h postinjection, the mice were euthanized, and major organs (heart, lungs, liver, kidneys, and spleen) were collected, washed with PBS, and analyzed for fluorescence intensity to assess nanoparticle targeting and retention in vivo.

### In vivo safety evaluation of membrane-coated nanoparticles

Six mice were randomly assigned to a control group (PBS injection) or a TP-EGCG treatment group (EGCG dose: 2 mg/kg). Mice received 100 μl of the corresponding formulation via tail vein injection every other day for 2 weeks. On day 15, mice were euthanized, and major organs (heart, liver, lungs, kidneys, and spleen) were collected, fixed in 4% paraformaldehyde, embedded in paraffin, and stained with H&E. Histopathological analysis was performed to assess the potential toxicity of nanoparticles on organ microstructure and evaluate their in vivo biocompatibility.

### Effect of membrane-coated nanoparticles on a CaOx mouse model

After 1 week of acclimation, a CaOx crystal deposition model was induced in mice using glyoxylic acid (75 mg/kg, intraperitoneal injection, once daily for 14 days) along with vitamin D₃ (1,500 IU/kg, subcutaneous injection, once weekly for 2 weeks). Mice in the experimental group were treated with TP-EGCG nanoparticles (EGCG dose: 2 mg/kg) via tail vein injection, while the control group received an equal volume of PBS (*n* = 6 per group). The intervention period matched the modeling period. Following the final treatment, mice were euthanized by cervical dislocation. Kidneys were collected and rinsed with PBS. One kidney was embedded in optimal cutting temperature compound (Beyotime, China), snap-frozen, and sectioned for DHE staining to assess ROS levels. The contralateral kidney was fixed in 4% paraformaldehyde. After dehydration, clearing, and paraffin embedding, tissue sections were prepared for H&E staining, Pizzolato’s staining to detect CaOx crystals, and IHC. The detailed procedure for Pizzolato’s staining is provided in the Supplementary Materials.

### Statistical analysis

#### Cohort analysis

Missing continuous variables were imputed using the median, and a separate “NA” category was created for categorical variables. Continuous variables were reported as mean ± standard deviation, and categorical variables were presented as frequency (percentage). Cumulative incidence was calculated using the Kaplan–Meier method, and Cox regression analysis results were reported as hazard ratios (HRs) with 95% confidence intervals (CIs).

#### MR analysis

The causal relationship between tea consumption and kidney stones was assessed using 4 methods: the inverse variance weighted (IVW) method, the MR-Egger method, the weighted median method, and the weighted mode method, with IVW as the primary method for estimating causal effects. Heterogeneity was assessed using Cochran’s *Q* test and funnel plots. A *P* value > 0.05 indicated no significant heterogeneity. Horizontal pleiotropy was assessed using the MR-Egger intercept test and MR-PRESSO global test. Leave-one-out analysis was performed to evaluate the influence of individual single-nucleotide polymorphisms (SNPs) on the overall results and assess the stability of the findings.

#### Cell and animal experiments

Categorical data were analyzed using the chi-square test and Fisher’s exact test. Numerical data were analyzed using *t* tests and one-way analysis of variance (ANOVA). Correlations between parameters were evaluated using Pearson correlation analysis. Each experiment was conducted at least 3 times.

All statistical analysis and visualization were performed using GraphPad Prism 10 and R software. Statistical significance was defined as *P* < 0.05.

## Results

### Tea consumption as a protective factor against kidney stone formation

Overall, 481,393 participants from UKB were included in this prospective cohort analysis. During a mean follow-up duration of 12.6 years, a total of 5,948 new cases of kidney stones were identified. The baseline characteristics are presented in Table S6. The HRs for kidney stone incidence were evaluated using 3 Cox proportional hazards models. As shown in Fig. 1B, compared to nondrinkers, both moderate and heavy tea drinkers demonstrated reduced risk of kidney stones. The cumulative incidence curves revealed that lower risk observed in participants with higher levels of tea consumption, and the protective effect was more pronounced over longer follow-up periods (Fig. 1C).

We further used MR analysis to assess the casual relationship between tea consumption and kidney stones (Fig. 1D and E). A total of 20 SNPs were included as IVs for tea consumption; the detailed information of SNPs is presented in Table S7. As shown in Fig. 1F and G, across multiple MR methods (IVW, MR-Egger, weighted median, and weighted mode), tea consumption was consistently associated with a lower risk of kidney stones. The IVW method demonstrated an OR of 0.45 (95% CI: 0.32 to 0.62). Sensitivity analyses showed no evidence of heterogeneity or horizontal pleiotropy (*P* > 0.05, Table S8). Leave-one-out analysis revealed that our MR results are robust (Fig. S1).

### EGCG attenuated COM-induced injury of renal tubular epithelial cells

**Fig. 2.**
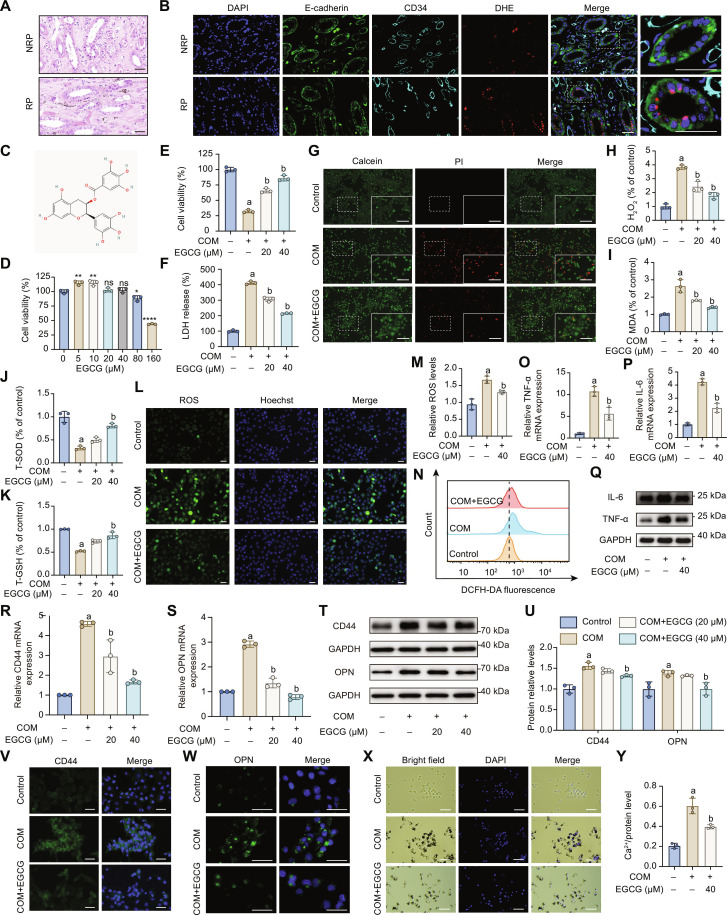
EGCG alleviated COM-induced HK-2 cell injury and crystal adhesion in vitro. (A) Von Kossa staining of NRP and RP tissues. NRP, normal renal papillae; RP, Randall’s plaque. (B) Dihydroethidium (DHE) staining and immunofluorescence analysis of kidney tissues showing oxidative stress (DHE), epithelial marker (E-cadherin), and endothelial marker (CD34) in RP (*n* = 6) versus NRP (*n* = 6) groups. (C) Structural formula of EGCG. (D) CCK-8 assay for HK-2 cells treated with different concentrations of EGCG for 24 h; *n* = 3. (E) CCK-8 assay was used to evaluate cell viability of HK-2 cells treated with COM crystals (100 μg/ml) with or without EGCG cotreatment for 24 h. (F) LDH release assay was employed to assess the cell death rate of HK-2 cells treated with COM crystals with or without EGCG cotreatment for 24 h. (G) Calcein/PI assay was used to stain viable and dead cells. (H to K) Assessment of oxidative and antioxidative indicators: H_2_O_2_ (H), MDA (I), T-SOD (J), and T-GSH (K) levels. (L to N) Total ROS levels detected using DCFH-DA (10 μM) and measured by fluorescence microscopy (L and M) and flow cytometry (N). (O to Q) qRT-PCR (O and P) and Western blot (WB) (Q) analyses of inflammatory factor (TNF-α and IL-6) expression in HK-2 cells incubated with COM crystals with or without EGCG treatment. (R to W) qRT-PCR (R and S), WB (T and U), and immunofluorescence (V and W) analyses of common stone-related protein (CD44 and OPN) expression in HK-2 cells incubated with COM crystals with or without EGCG treatment. (X) Observation of COM crystals adhesion on HK-2 cells surfaces using inverted fluorescence microscopy. (Y) Measurement of calcium ion concentration after 24-h dissolution of extracellular COM crystals in 2 mol/l HCl and normalization by total protein concentration. Scale bar = 50 μm. The results were shown as mean ± SD of 3 individual experiments. One-way ANOVA followed by Tukey’s post hoc test was performed for multiple-group comparisons. a: *P* < 0.05 vs. the Control group; b: *P* < 0.05 vs. the COM group.

Considering that CaOx stones account for approximately 80% of kidney stones [8] and that Randall’s plaques serve as niduses for CaOx nephrolithiasis [25], we initially focused on the pathomorphology of Randall’s plaques. Von Kossa staining showed calcium salt deposition predominantly localized in the peri-tubular regions within Randall plaque tissues (Fig. 2A). Furthermore, utilizing DHE coupled with immunofluorescence analyses, we identified a significant elevation of oxidative stress in renal tubules adjacent to these calcium deposits (Fig. 2B), which supported that renal tubular epithelial injury contributed to Randall’s plaque formation. Additionally, hypercalciuria- or/and hyperoxaluria-associated COM crystals are identified as one of the key factors contributing to renal tubular epithelial injury [10]. Meanwhile, EGCG (Fig. 2C), the primary bioactive component in green tea, is believed to play a key role in its protective effect on nephrolithiasis. Therefore, we performed a coculture of EGCG, HK-2 cells, and COM for further investigation. Based on the results of the CCK-8 assay (Fig. 2D), when the EGCG concentration exceeded 80 μM, a significant decrease in cell viability was observed. Therefore, 20 and 40 μM EGCG were selected for subsequent experiments to ensure an effective dose with minimal impact on cell viability.

Exposure of HK-2 cells to COM significantly reduced cell viability, increased LDH release, and elevated red fluorescence representing dead cells, while EGCG treatment restored cell viability and decreased LDH release in a dose-dependent manner (Fig. 2E to G). The protective effects of EGCG against COM-induced oxidative stress in HK-2 cells were further evaluated. Intracellular ROS levels, assessed using DCFH-DA fluorescence, were significantly elevated by COM exposure but were markedly reduced by cotreatment with EGCG (Fig. 2L to N). Additionally, EGCG alleviated COM-induced oxidative stress by lowering levels of H_2_O_2_ and MDA, while enhancing the activities of antioxidant enzymes T-SOD and CAT (Fig. 2H to K).

EGCG also demonstrated anti-inflammatory properties by reducing the mRNA and protein levels of tumor necrosis factor-α (TNF-α) and interleukin-6 (IL-6) (Fig. 2O to Q). These cytokines are critical mediators of the inflammatory response and are associated with renal injury and the development of kidney stones [28]. Furthermore, EGCG suppressed the expression of stone-related markers CD44 and OPN at both mRNA and protein levels (Fig. 2R to U and Fig. S2), since CD44 may facilitate the adhesion of CaOx crystals to tubular epithelial cells through its interaction with the ligand OPN [29]. EGCG could also reduce crystal adhesion to the surface of HK-2 cells (Fig. 2X), which was further supported by the decreased Ca^2+^ protein levels observed in the EGCG treatment group (Fig. 2Y). Collectively, these findings suggested that EGCG mitigates COM-induced HK-2 cell injury through its antioxidative, anti-inflammatory, and anti-adhesion properties, highlighting its potential therapeutic role in preventing kidney stone formation.

### EGCG protected HK-2 cells from COM-induced injury by targeting GRP94

**Fig. 3.**
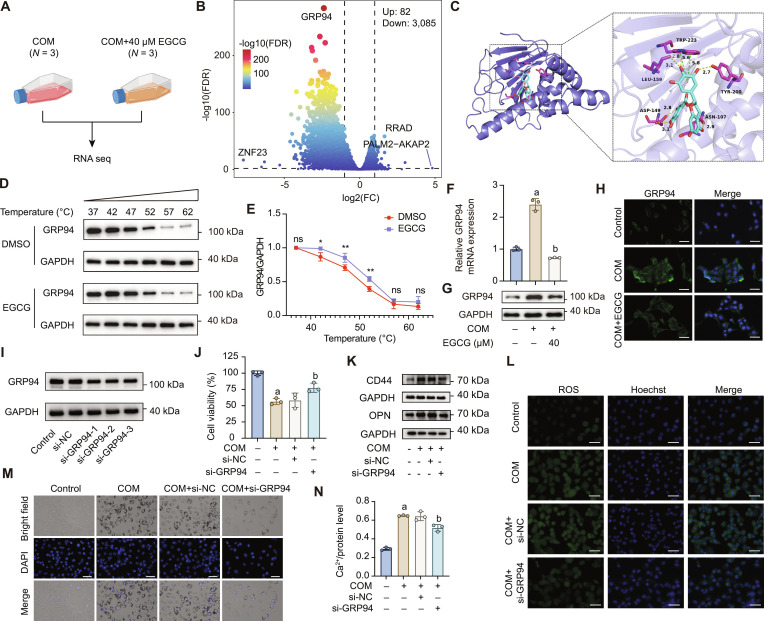
EGCG protects HK-2 cells from COM-induced injury by targeting GRP94. (A) RNA sequencing was performed on HK-2 cells exposed to COM crystals with or without EGCG (40 μM). (B) Volcano plot of screening differential expressed genes. (C) Molecular docking analysis of EGCG with GRP94 (affinity = −8.3 kcal/mol). (D and E) Cellular thermal shift assays of the GRP94 protein (*n* = 3). (F to H) qRT-PCR (F), WB (G), and immunofluorescence (H) analyses of GRP94 expression in HK-2 cells incubated with COM crystals with or without EGCG treatment (*n* = 3). (I) WB analysis to assess knockdown efficiency of siRNA for GRP94. (J) CCK-8 assay was used to assess cell viability of HK-2 cells treated with COM crystals with or without GRP94 knockdown (*n* = 3). (K) WB analysis to evaluate the expression of common stone-related protein (CD44 and OPN) in HK-2 cells incubated with COM crystals with or without GRP94 knockdown. (L) Total ROS levels detected using DCFH-DA (10 μM) and measured by fluorescence microscopy. (M) Observation of COM crystals adhesion on HK-2 cells surfaces using inverted fluorescence microscopy. (N) Measurement of calcium ion concentration after 24-h dissolution of extracellular COM crystals in 2 mol/l HCl and normalization by total protein concentration (*n* = 3). Scale bar = 50 μm. The results were shown as mean ± SD of 3 individual experiments. Student’s *t* test was performed for 2-sample comparisons and one-way ANOVA followed by Tukey’s post hoc test was performed for multiple-group comparisons. ns: *P* > 0.05; *P < 0.05; **P < 0.01; a: *P* < 0.05 vs. the Control group; b: *P* < 0.05 vs. the COM group (with or without si-NC).

To identify the target genes involved in the protective effects of EGCG, we performed transcriptomic analysis using RNA sequencing. RNA sequencing was conducted on HK-2 cells exposed to COM with or without EGCG (40 μM), as shown in Fig. 3A. The subsequent analysis revealed DEGs, as highlighted in Fig. 3B, with 82 genes up-regulated and 3,085 genes down-regulated upon EGCG treatment. The heatmap of DEGs is shown in Fig. S3. We selected the 3 most significantly DEGs for molecular docking analysis with EGCG, including GRP94, HIF1A, and HSP90-α (Fig. S4A and B). Among these, GRP94 exhibited the highest binding affinity with EGCG, with a binding energy of −8.3 kcal/mol, indicating a strong interaction (Fig. 3C). Cellular thermal shift assay (CETSA) further confirmed that EGCG increased the thermal stability of GRP94, which suggested that EGCG could directly interact with GRP94 (Fig. 3D and E). Next, we validated the expression of GRP94 in HK-2 cells. As shown in Fig. 3F and G, COM exposure significantly increased GRP94 mRNA and protein levels, which were markedly suppressed by EGCG treatment. These findings were further corroborated by immunofluorescence analysis (Fig. 3H), suggesting that EGCG effectively down-regulated GRP94 expression in COM-exposed HK-2 cells.

To explore the role of GRP94 in COM-induced HK-2 cell injury, GRP94 was silenced using siRNA, and the knockdown efficiency was validated at both the mRNA and protein levels (Fig. 3I and Fig. S5A and B). As shown in Fig. 3J and K, GRP94 knockdown significantly improved cell viability and reduced the expression of stone formation markers CD44 and OPN. Additionally, GRP94 silencing mitigated oxidative stress, as evidenced by reduced intracellular ROS levels (Fig. 3L). Bright-field imaging and calcium ion concentration measurements further confirmed that GRP94 knockdown effectively reduced extracellular crystal adhesion (Fig. 3M and N).

### EGCG attenuates COM-induced HK-2 cell injury by inhibiting the GRP94/PI3K/AKT axis

**Fig. 4.**
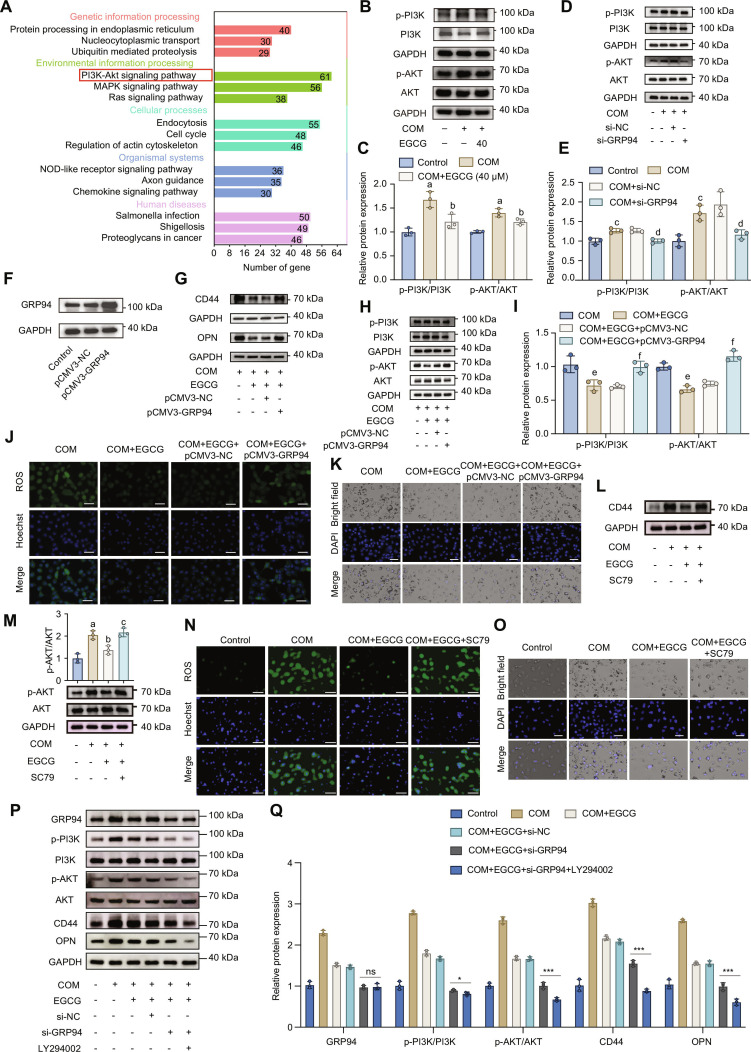
EGCG attenuated COM-induced HK-2 cells injury by inhibiting GRP94/PI3K/AKT axis. (A) KEGG pathway enrichment analysis of DEGs. (B and C) WB analysis was used to examine the activation of PI3K and AKT in HK-2 cells incubated with COM crystals with or without EGCG treatment (*n* = 3). (D and E) WB analysis was used to examine the activation of PI3K and AKT in HK-2 cells incubated with COM crystals with or without GRP94 knockdown (*n* = 3). (F) The overexpression efficiency of GRP94 was confirmed using WB analysis. (G) WB analysis to evaluate the expression of common stone-related protein (CD44 and OPN). (H and I) WB analysis was used to examine the activation of PI3K and AKT (*n* = 3). (J) Total ROS levels measured by fluorescence microscopy. (K) Observation of COM crystals adhesion on HK-2 cells surfaces using inverted fluorescence microscopy. (L) WB analysis to evaluate the expression of CD44. (M) WB analysis was used to examine the activation of AKT (*n* = 3). (N) Total ROS levels measured by fluorescence microscopy. (O) Observation of COM crystals adhesion on HK-2 cells surfaces using inverted fluorescence microscopy. (P and Q) WB analysis of GRP94, p-PI3K, PI3K, p-AKT, AKT, CD44, and OPN expression in HK-2 cells under different treatments (*n* = 3). Scale bar = 50 μm. The results were shown as mean ± SD of 3 individual experiments. One-way ANOVA followed by Tukey’s post hoc test was performed for multiple-group comparisons. a: *P* < 0.05 vs. the Control group; b: *P* < 0.05 vs. the COM group; c: *P* < 0.05 vs. the Control group; d: *P* < 0.05 vs. the COM + si-NC group; e: *P* < 0.05 vs. the COM group; f: *P* < 0.05 vs. the COM + EGCG + pCMV3-NC group; ns, not significant; ***P < 0.001.

As shown in Fig. 4A, KEGG pathway enrichment analysis of DEGs revealed significant enrichment in the phosphatidylinositol 3-kinase/protein kinase B (PI3K/AKT) signaling pathway, suggesting its involvement in the protective effect of EGCG on COM-induced HK-2 cell injury. WB analysis (Fig. 4B and C) demonstrated that COM exposure significantly increased the phosphorylation levels of PI3K and AKT, indicating activation of the PI3K/AKT signaling pathway. EGCG treatment markedly reduced the phosphorylation levels of both PI3K and AKT, as quantified in Fig. 4C. To confirm the role of GRP94 in PI3K/AKT signaling, GRP94 was knocked down in HK-2 cells using siRNA. As shown in Fig. 4D and E, GRP94 knockdown significantly decreased the phosphorylation levels of PI3K and AKT induced by COM. These results indicate that GRP94 is an upstream regulator of the PI3K/AKT pathway in COM-induced injury.

To further explore the interaction between GRP94 and the PI3K/AKT pathway, GRP94 was overexpressed using pCMV3-GRP94, and the overexpression efficiency was validated (Fig. 4F and Fig. S4C and D). WB analysis (Fig. 4H and I) demonstrated that GRP94 overexpression rescued PI3K and AKT phosphorylation levels that were suppressed by EGCG, indicating that GRP94 mediated EGCG’s effects on the PI3K/AKT pathway. Additionally, GRP94 overexpression reversed the inhibitory effects of EGCG on the expression of key stone-related markers, CD44 and OPN (Fig. 4G), ROS levels (Fig. 4J), and crystal adhesion (Fig. 4K).

To validate the role of the PI3K/AKT pathway in COM-induced injury, HK-2 cells were treated with SC79, an AKT agonist. WB analysis (Fig. 4M) confirmed that SC79 restored the phosphorylation levels of AKT, even in the presence of EGCG. Notably, SC79 treatment reversed EGCG’s protective effects by increasing ROS levels (Fig. 4N), as well as restoring CD44 expression (Fig. 4L) and crystal adhesion (Fig. 4O). In contrast, inhibition of PI3K with LY294002 further potentiated the effects of EGCG, leading to greater suppression of p-AKT, CD44, and OPN, and concomitantly reducing ROS production and extracellular crystal adhesion (Fig. 4P and Q and Fig. S6). These findings confirm that EGCG exerts its renoprotective effects against COM-induced injury primarily through modulation of the GRP94/PI3K/AKT signaling pathway.

### Activation of the GRP94/PI3K/AKT axis in RP tissues

**Fig. 5.**
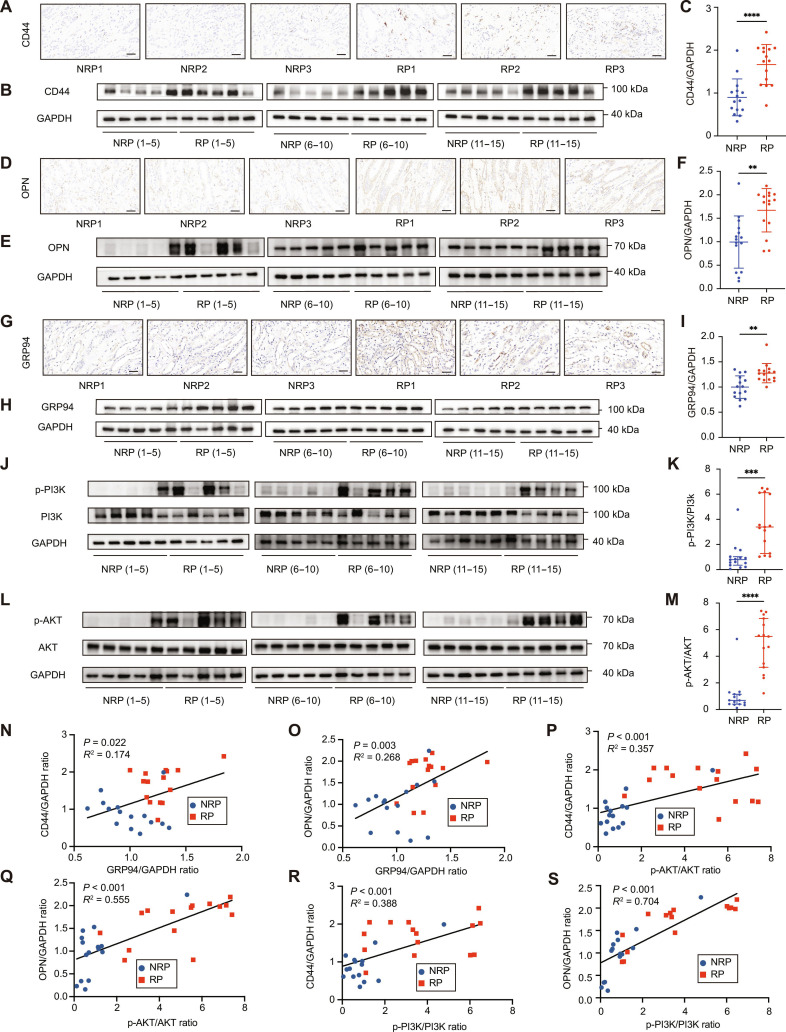
Activation of the GRP94/PI3K/AKT axis in Randall’s plaque tissues. (A to C) Immunohistochemical staining (A) and WB analysis (B and C) showed the expression levels of CD44 in the NRP and RP tissues. (D to F) Immunohistochemical staining (D) and WB analysis (E and F) showed the expression levels of OPN in the NRP and RP tissues. (G and H) Immunohistochemical staining (G) and WB analysis (H and I) showed the expression levels of GRP94 in the NRP and RP tissues. (J to M) WB analysis evaluated the activation of PI3K (J and K) and AKT (L and M) in the NRP and RP tissues. (N and O) Correlation between relative GRP94 protein expression and OPN and CD44 levels determined by WB in NRP and RP tissues. (P to S) Correlation between the expression of PI3K/AKT pathway proteins and OPN and CD44 levels determined by WB in NRP and RP tissues. Scale bar = 50 μm. NRP: *N* = 15; RP: *N* = 15. Student’s *t* test was performed for 2-sample comparison. **P < 0.01; ***P < 0.001; ****P < 0.0001.

We further evaluated the expression of above key molecules in the RP tissues. Immunohistochemical staining (Fig. 5A, D, and G) and WB analysis (Fig. 5B, E, and H) showed significantly higher expression levels of CD44, OPN, and GRP94 in the RP group compared to the NRP group, which was supported by the quantification of WB (Fig. 5C, F, and I) and immunohistochemical staining (Fig. S7A to C).

We further assessed the activation of the PI3K/AKT pathway in RP tissues. WB analysis revealed significantly higher phosphorylation levels of PI3K and AKT in the RP group compared to the NRP group (Fig. 5J and L), with quantification confirming increased p-PI3K/PI3K (Fig. 5K) and p-AKT/AKT (Fig. 5M) ratios in the RP group. These results indicate activation of the PI3K/AKT signaling pathway in patients with RP.

Correlation analyses were performed to explore the relationships between GRP94, PI3K/AKT signaling pathway, and the expression of stone-related markers. GRP94 expression was positively correlated with CD44 (Fig. 5N) and OPN (Fig. 5O) levels. Furthermore, the activation of the PI3K/AKT pathway (p-PI3K/PI3K and p-AKT/AKT ratios) was positively correlated with CD44 (Fig. 5P and R) and OPN (Fig. 5Q and S) expression. These results highlight the potential role of GRP94-mediated PI3K/AKT pathway activation in the pathogenesis of RP.

### TP-EGCG nanoparticles reduced crystal deposition in the kidney stone mouse model

**Fig. 6.**
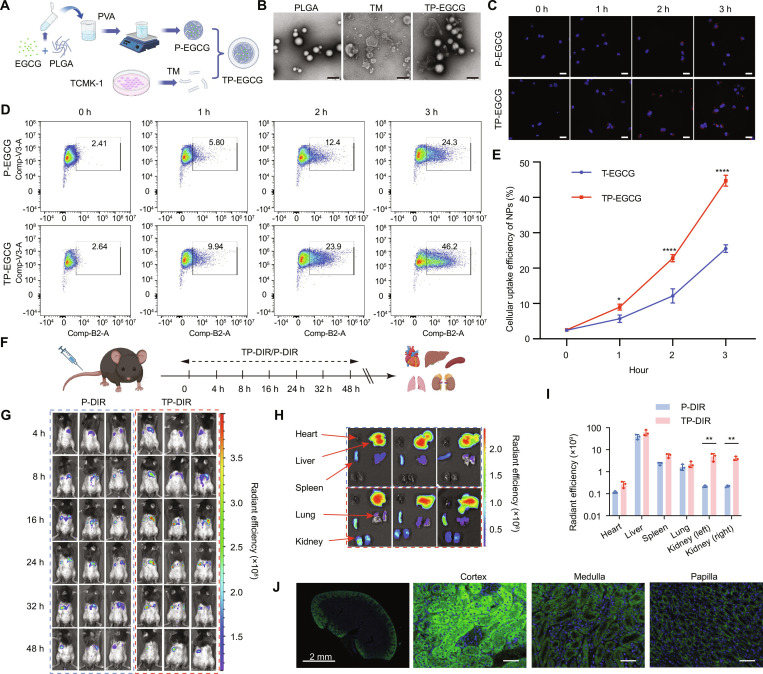
Membrane-coated nanoparticles enhance cellular uptake and renal targeting of EGCG both in vitro and in vivo. (A) Schematic of TP-EGCG prepared. (B) SEM image of PLGA, TM, and TP-EGCG. Scale bar = 200 nm. (C) Confocal laser scanning microscopy (CLSM) images showing the intracellular uptake of P-EGCG and TP-EGCG in TCMK-1 cells at different time points (0 to 3 h). (D and E) Flow cytometry analysis (D) and quantitative analysis (E) of cellular uptake efficiency of P-EGCG and TP-EGCG over time (*n* = 3). (F) Schematic diagram of animal experiments to assess the in vivo biodistribution of TP-NPs. (G) In vivo fluorescence imaging at various time points (4 to 48 h) showing the biodistribution of P-DIR and TP-DIR in mice. (H and I) Ex vivo fluorescence imaging and quantitative analysis of major organs (heart, liver, spleen, lung, and kidneys) at 48 h postinjection (*n* = 3). (J) Distribution of TP-DIR in kidney tissue sections visualized by fluorescence microscopy. Scale bar = 50 μm. Student’s *t* test was performed for 2-sample comparison. *P < 0.05, **P < 0.01, ***P < 0.001, ****P < 0.0001.

To enhance the bioavailability of EGCG, TP-EGCG nanoparticles were developed using PLGA encapsulation combined with TCMK-1 cell membranes for targeted kidney delivery (Fig. 6A). TEM images (Fig. 6B) showed the morphology of PLGA, TM, and TP-EGCG. The membrane-coated nanoparticles exhibited a characteristic core–shell structure, confirming successful nanoparticle synthesis. Dynamic light scattering further demonstrated that TP-EGCG had a uniform size distribution of approximately 180 to 200 nm with a low PDI (<0.2), and the zeta potential was moderately positive (+6 to +8 mV), indicating good colloidal stability (Fig. S8A to C). The encapsulation efficiency and drug loading were ~45% and ~1.2%, respectively (Fig. S8E). In vitro release assays revealed a sustained-release profile, with cumulative EGCG release reaching ~75% at pH 7.4 and ~90% at pH 6.5 within 14 days (Fig. S8D). These results confirm that TP-EGCG possesses favorable physicochemical characteristics for subsequent biological evaluation. Fluorescent imaging (Fig. 6C) and flow cytometry analysis (Fig. 6D and E) demonstrated that TP-EGCG exhibited higher cellular uptake efficiency compared to P-EGCG in TCMK-1 cells over 0 to 3 h. These findings indicate enhanced cellular delivery of EGCG through TP-EGCG nanoparticles.

To evaluate the in vivo biodistribution of the membrane-coated nanoparticles, fluorescently labeled TP-DIR nanoparticles were administered to mice, and their distribution was tracked using live imaging and ex vivo organ analysis (Fig. 6F to J). In vivo imaging of mice (Fig. 6G) showed that TP-DIR preferentially accumulated in the kidney, with higher fluorescence intensity compared to P-DIR at 48 h postinjection. Ex vivo imaging of organs (Fig. 6H and I) confirmed that TP-DIR exhibited enhanced kidney targeting, and the distribution of TP-DIR in the kidney tissue is shown in Fig. 6J.

Biochemical analysis revealed that serum creatinine, blood urea nitrogen, alanine aminotransferase, and aspartate aminotransferase levels were not significantly altered by TP-EGCG treatment compared with controls (Fig. S9A), supporting the systemic safety of the TP-EGCG. Additionally, H&E staining was used to perform histopathological analysis of 5 major mouse organs (lung, heart, liver, spleen, and kidney) to further assess the biosafety of TP-EGCG. The results showed intact tissue structures in all organs, with no signs of inflammatory cell infiltration or tissue damage (Fig. S9B).

**Fig. 7.**
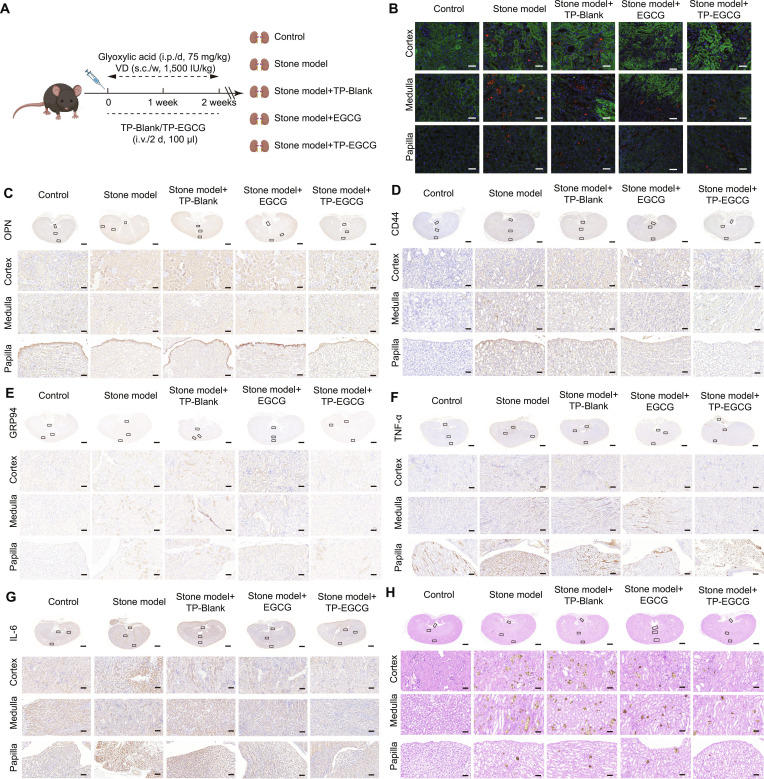
TP-EGCG nanoparticles (NPs) reduced crystal deposition in the kidney stone mouse model. (A) Schematic diagram of animal experiments. The kidney stone mouse model was established through daily intraperitoneal injections of glyoxylate (75 mg/kg/day) combined with subcutaneous injections of vitamin D (1,500 IU/kg) once a week for 2 weeks. The TP-Blank/TP-EGCG-treated group was given 100 μl once every 2 days, through tail intravenous injections. (B) DHE staining to detect reactive oxygen species (ROS) generation in mouse kidney tissues. (C to G) Immunohistochemical staining of kidney from the different groups (Control, Stone model, Stone model + TP-Blank, and Stone model + TP-EGCG): OPN (C), CD44 (D), GRP94 (E), TNF-α (D), and IL-6 (E). (H) CaOx crystal deposition in TP-EGCG treated kidney stone mouse model was detected by Pizzolato’s and H&E staining. Scale bar = 50 μm. **P < 0.01.

To evaluate the therapeutic potential of TP-EGCG, a glyoxylic acid-induced kidney stone mouse model was established (Fig. 7A). DHE staining revealed increased ROS generation in the renal tissues of the stone model group, while TP-EGCG treatment significantly reduced ROS levels (Fig. 7B). Immunohistochemical staining revealed that the expression of OPN (Fig. 7C), CD44 (Fig. 7D), and GRP94 (Fig. 7E) was markedly elevated in stone model mice but significantly reduced in the TP-EGCG-treated group compared to the TP-Blank group (Fig. S10A to C). These results indicated that TP-EGCG suppressed the expression of key markers involved in kidney stone formation. Moreover, the expression of the inflammatory cytokines TNF-α and IL-6 was also elevated in the stone model group and significantly decreased after TP-EGCG administration (Fig. 7F and G and Fig. S10D and E), further supporting the anti-inflammatory effect of the TP-EGCG in vivo.

H&E staining and Pizzolato’s staining (Fig. 7H) showed extensive CaOx deposition in the cortex, medulla, and papilla of stone model mice. These pathological changes were alleviated in the TP-EGCG-treated group, highlighting its protective effects against kidney stones formation. Additionally, polarized light microscopy (Fig. S11) demonstrated that TP-EGCG treatment reduced CaOx crystal deposition in renal tissues, providing additional evidence of its therapeutic potential.

## Discussion

This study revealed the potential association between tea consumption and reduced kidney stone risk, as well as the underlying mechanisms, through a multidimensional investigation. A population-based cohort analysis using UKB data showed that tea consumption was associated with a reduced risk of kidney stones. MR analysis further demonstrated that genetically predicted tea consumption reduced the risk of kidney stone formation, offering a new explanation for the conflicting findings of observational studies on tea consumption and kidney stone risk. In vitro experiments demonstrated that EGCG, the main active component of green tea, protects cells through multiple pathways, including regulation of oxidative stress, inflammation, and crystal adhesion. Mechanistic studies revealed that EGCG reduces CaOx crystal-induced cell damage by regulating the PI3K/AKT pathway through GRP94. Animal experiments further confirmed the feasibility of the kidney-targeted nanoparticle delivery system in enhancing the therapeutic effects of EGCG, providing a new strategy and experimental basis for the prevention and treatment of kidney stones with EGCG.

Previous observational studies have not reached a clear consensus on the relationship between tea consumption and kidney stone risk. A cross-sectional study conducted in Northern China found that tea consumption was associated with an increased risk of kidney stones [30], while a prospective cohort study in middle-aged and elderly Chinese populations reported a negative association between green tea intake and the incidence of kidney stones [4]. In the present study, analysis of the latest UKB cohort data further supports a significant association between increased tea consumption and reduced kidney stone risk.

To overcome the limitations of observational studies, an MR analysis was conducted to infer causality. The results showed that increased tea consumption has a potential protective effect against kidney stones, consistent with the team’s previous meta-analysis findings [31]. Evidence suggests that the protective mechanisms of tea consumption may involve multiple pathways. Increased tea intake enhances overall fluid consumption, promotes urine production, and reduces the supersaturation of urinary crystals. Caffeine, as a natural diuretic, increases urine flow rate and regulates the expression of adhesion proteins on renal tubular epithelial cells, thereby inhibiting the abnormal retention of CaOx crystals [32]. Notably, black tea intake has been shown to significantly increase urinary citrate excretion, which acts as a crystallization inhibitor by preventing CaOx crystal nucleation [33]. Polyphenols in tea scavenge ROS, reducing oxidative damage to renal tubules induced by CaOx crystals [34]. Overall, these mechanisms collectively contribute to a plausible biological explanation for the observed protective association between tea consumption and kidney stone risk.

CaOx stones are the most common type of kidney stones, with COM crystals being the primary crystalline form and the most lithogenic (stone-forming) hydrated form [35]. The pathogenesis of CaOx stones involves urinary supersaturation, crystal nucleation, growth, aggregation, and interaction with renal tubular epithelial cells, leading to crystal attachment and retention in the renal tubules [9]. The interaction between CaOx crystals and renal tubular epithelial cells plays a critical role in this process. CaOx crystals stimulate renal tubular epithelial cells to produce large amounts of ROS, triggering oxidative stress and cellular damage. Excessive ROS activate the NF-κB signaling pathway, inducing inflammatory factors and stone-related proteins, which further damage epithelial cells [36]. Damaged epithelial cells not only increase their adhesion to CaOx crystals but also produce membrane debris during injury, which may serve as a core for CaOx stone formation, further promoting stone progression and development [37]. Similar inflammatory mechanisms have also been observed in other crystal-induced disorders. For example, monosodium urate crystals in gout activate NF-κB and the NLRP3 inflammasome, leading to persistent inflammation and tissue injury [38]. These parallels suggest that different types of pathogenic crystals may converge on common molecular pathways, reinforcing the rationale for targeting oxidative stress and inflammation as potential strategies to prevent crystal-associated diseases, including CaOx nephrolithiasis. Therefore, reducing oxidative stress levels and alleviating inflammatory responses may be effective strategies for preventing kidney stone formation.

Growing evidence suggests that natural bioactive compounds have remarkable potential in preventing kidney stones. EGCG, the most biologically active polyphenol in green tea, has been extensively studied. This study found that EGCG reduces ROS production induced by COM crystals and improves oxidative stress markers, consistent with previous findings. Previous studies have shown that EGCG directly inhibits ROS production and enhances cellular antioxidant capacity by activating the expression of antioxidant enzymes through the Keap1–Nrf2–ARE pathway [39]. Moreover, EGCG reduces ROS-mediated inflammatory responses by inhibiting the NF-κB signaling pathway [39]. In this study, we also found that EGCG reduces COM crystal-induced inflammation markers, further supporting its anti-inflammatory and antioxidant effects. Crystal adhesion is critical in kidney stone formation, with CD44 and OPN being the most common adhesion molecules [40,41]. This study showed that OPN and CD44 expression was significantly elevated in renal tissue from stone patients. In vitro experiments demonstrated that COM crystals promote CD44 and OPN expression, which was significantly reduced by cotreatment with EGCG. Light microscopy showed that fewer crystals adhered to the cell surface, suggesting that EGCG effectively inhibits crystal adhesion and prevents stone formation. Beyond these local renal actions, EGCG may also exert systemic benefits through the gut–kidney axis. Emerging clinical evidence indicates that alterations in gut microbiota composition are associated with renal outcomes, including fibrosis in peritoneal dialysis patients [42]. As EGCG has been shown to modulate the gut microbiota [43], it is plausible that part of its long-term renoprotective effect could involve microbiota-mediated pathways. Although this mechanism was not directly investigated in the present study, it warrants further investigation in future research.

To explore the target of EGCG, this study conducted transcriptome analysis, which suggested that the GRP94/PI3K/AKT pathway may be the key mechanism underlying the protective effects of EGCG. GRP94, a member of the HSP90 family, is located in the ER and encoded by the HSP90B1 gene, sharing approximately 50% homology with cytoplasmic HSP90 [44]. In our study, the expression of GRP94 was elevated in cell models, animal models, and clinical specimens. Previous studies have shown that cytoplasmic HSP90 can translocate to the cell membrane, where it serves as a receptor for COM crystals [45]. EGCG has been shown to inhibit the membrane localization of HSP90, thereby reducing COM crystal adhesion [45]. As an important member of the HSP90 family, GRP94 may be similarly inhibited by EGCG. Previous studies have shown that EGCG acts as an HSP90 inhibitor, exhibiting significant antitumor activity [46,47]. This study confirmed that EGCG reduces COM crystal-induced GRP94 expression. Molecular docking and CETSA assays confirmed that EGCG directly binds to GRP94, thereby inhibiting its function.

Previous studies have shown that the PI3K/AKT pathway is activated in CaOx stones [48,49]. COM crystals increase ROS production through NADPH oxidase, leading to the activation of the PI3K/AKT, NF-κB, and TGF-β (transforming growth factor-β) pathways, which promote inflammation and fibrosis [48]. Oxalate also enhances crystal-cell adhesion and regulates macrophage metabolism and polarization through the JPT2/PI3K/AKT pathway [49]. This study found that the PI3K/AKT pathway was significantly activated in the COM crystal treatment group, whereas cotreatment with EGCG suppressed this activation, suggesting that the protective effects of EGCG may depend on inhibition of the PI3K/AKT pathway.

**Fig. 8.**
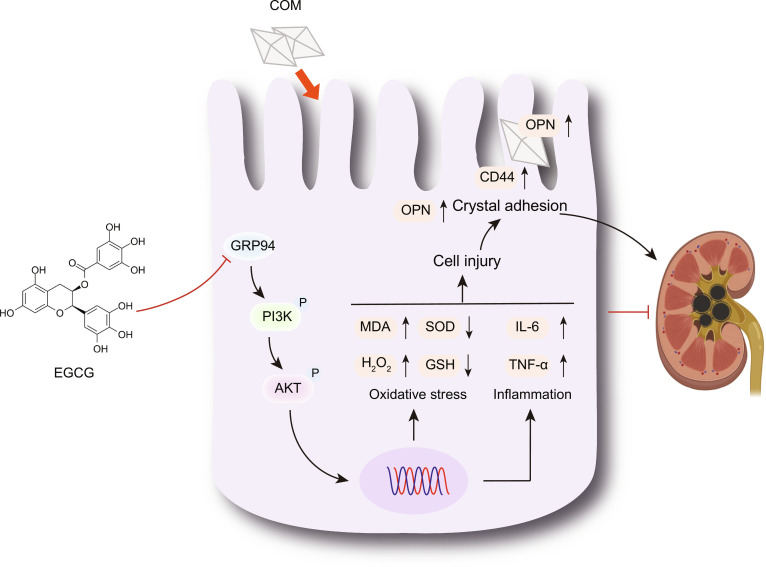
Schematic illustration of the protective effects and underlying mechanisms of EGCG against COM crystal-induced injury in renal tubular epithelial cells. EGCG may protect cells by inhibiting the GRP94/PI3K/AKT axis, thereby reducing COM crystal-induced oxidative stress, inflammatory responses, and crystal adhesion.

To further confirm whether EGCG acts through the GRP94/PI3K/AKT axis, we first silenced GRP94 expression using siRNA. GRP94 silencing improved COM crystal-induced cell injury, reduced ROS production, down-regulated OPN and CD44 expression, and suppressed PI3K/AKT pathway activation. Next, we overexpressed GRP94 to assess the protective effects of EGCG under GRP94 overexpression conditions. GRP94 overexpression weakened the protective effects of EGCG, enhanced crystal adhesion, and reactivated the PI3K/AKT pathway. Finally, AKT activation with SC79 diminished EGCG’s protective effects. Thus, EGCG exerts protective effects by inhibiting the GRP94/PI3K/AKT axis, thereby reducing oxidative stress, inflammation, and crystal adhesion (Fig. 8).

Due to EGCG’s low bioavailability and poor stability, nanoparticle encapsulation has been increasingly explored to overcome these limitations. Previous research has shown that synthetic EGCG-PLGA nanoparticles exhibit stronger antioxidant capacity than free EGCG [19]. In this study, we constructed TCMK-1 cell membrane-coated EGCG-PLGA nanoparticles (TP-EGCG) to enhance bioavailability and renal targeting. Animal experiments evaluated targeting ability and safety in mice, with functional validation in a mouse CaOx crystal deposition model. TP-EGCG injection significantly reduced renal CaOx crystals, lowered CD44 and OPN expression levels, and down-regulated GRP94 expression. Thus, TP-EGCG offers a novel targeted strategy for preventing and treating CaOx stones.

Although this study explored the relationship between tea consumption and kidney stones from multiple angles, some limitations remain. First, the specific type of tea and kidney stones was not clearly defined in the cohort study and MR analysis, making it impossible to evaluate the differential effects of various types of tea on CaOx kidney stone formation, though CaOx stones account for ~80% of nephrolithiasis [8]. It should be acknowledged that different tea types vary markedly in their composition. Green tea is typically richer in catechins such as EGCG, which exhibit antioxidant and anti-inflammatory activities and may protect against kidney stone formation. By contrast, black and oolong teas contain lower catechin concentrations but relatively higher oxalate levels, which could contribute to lithogenic risk. These compositional differences highlight the importance of distinguishing tea types in future research to better clarify their respective risks and benefits in relation to nephrolithiasis. In addition, the study population consisted entirely of Europeans, raising uncertainty about whether the findings can be generalized to populations with different ethnic backgrounds, dietary habits, or tea formulations. Validation in more diverse cohorts is needed to strengthen the generalizability of our results. Second, our study demonstrated that EGCG reduces COM-induced injury primarily through its anti-inflammatory and antioxidant effects, thereby protecting HK-2 cells and decreasing renal CaOx crystal deposition. Given that ER stress is also crucial in CaOx stone formation [50], we examined the ATF6 pathway. COM treatment markedly increased ATF6 expression, but EGCG cotreatment did not significantly change ATF6 levels. These results indicate that EGCG’s protective effects are unlikely to be mediated by ATF6-dependent signaling. Instead, EGCG may alleviate ER stress-related apoptosis through alternative pathways, which merits further investigation in future studies. Third, while we provided detailed physicochemical characterization of TP-EGCG and confirmed that it did not cause elevations in biochemical indicators in animal experiments, further studies are still needed to assess long-term stability, biodistribution, and potential off-target effects under different dosing regimens. Moreover, the EGCG concentrations applied in vitro (20 to 40 μM) exceed the plasma levels typically achieved through regular tea consumption (1 μM). Although renal accumulation and nanoparticle delivery may help bridge this gap, caution is warranted when extrapolating these doses to humans, and pharmacokinetic as well as dose-finding studies are required to establish physiologically relevant exposure levels. Finally, although in animal studies we observed that TP-EGCG effectively reduced CaOx crystal formation, its long-term safety, pharmacokinetics, and efficacy in humans remain uncertain. Future clinical studies are therefore warranted to validate its protective effects, determine optimal dosing strategies, and further assess the translational potential of EGCG-loaded nanoparticles.

## Conclusion

This study suggests that tea consumption may reduce the risk of kidney stones. EGCG in tea protects renal tubular epithelial cells from CaOx crystal-induced damage by inhibiting the GRP94/PI3K/AKT axis. Cell membrane-coated EGCG-PLGA nanoparticles enhance renal targeting and reduce CaOx deposition, offering new insights for developing EGCG-based strategies for the prevention of CaOx stones.

## Ethical Approval

This study was approved by the Xiangya Hospital Ethics Committee (Approval No. 202103089) and the Institutional Experimental Animal Committee of Central South University (Approval Nos. CSU-2022-0022 and CSU-2022-0454). Written informed consent was obtained from all patients prior to surgery for sample collection and anonymous use of their clinical data for publication purposes.

## Data Availability

The data that support the findings of this study are available from the corresponding authors upon reasonable request.
