# Supplementary material for: Epigallocatechin Gallate Attenuates CaOx Crystal-Induced Renal Tubular Injury to Inhibit CaOx Nephrolithiasis via GRP94/PI3K/AKT Signaling
Source: Biomater Res. 2025 Nov 17;29:0271. doi: 10.34133/bmr.0271 (PMC12620625; doi:10.34133/bmr.0271)
Supplement: Supplementary 1 — Figs. S1 to S11 Tables S1 to S8 [file bmr.0271.f1.zip › Supplementary materials.docx]

**Supplementary Materials**

**Supplementary Methods:**

1.1 Selection of Genetic Instrumental Variables (IVs)

Genetic variants associated with tea consumption were derived from the second-round GWAS data of the UK Biobank (curated by the Neale Lab), comprising 349,376 individuals. The selection process for IVs (single nucleotide polymorphisms, SNPs) involved the following steps. SNPs significantly associated with tea consumption were identified using a genome-wide significance threshold of P < 5×10⁻⁸. To ensure independence among selected SNPs, clustering analysis was performed with a window size of 10,000 kb and a linkage disequilibrium threshold of R² < 0.001, yielding 20 independent SNPs. The strength of each instrumental SNP was assessed using the F-statistic (F = Beta²/SE²), and only SNPs with F ≥ 10 were retained to reduce the risk of weak instrument bias[1].

1.2 Cell Viability Assay

Cell viability was assessed using the Cell Counting Kit-8 (CCK-8) assay. HK-2 cells were plated at a density of 6×10³ cells per well in a 96-well plate and incubated at 37°C for 24 hours to allow for adherence. The cells were then treated according to the experimental design and further incubated for another 24 hours. After treatment, the culture medium was removed and replaced with MEM supplemented with 10% (v/v) CCK-8 reagent. The plate was incubated at 37°C for 1 hour, after which the optical density (OD) was measured at 450 nm using a multimode microplate reader.

1.3 Lactate Dehydrogenase (LDH) Release Assay

Cell damage was assessed using the LDH release assay. HK-2 cells were resuspended, evenly mixed, and seeded into 12-well plates. The cells were incubated at 37°C with 5% CO₂ and saturated humidity for 24 hours to allow for proper adherence. Cells were then treated according to the experimental design. Following treatment, 1 mL of supernatant was collected from each group, including a blank control, and transferred to a 1.5 mL Eppendorf (EP) tube. The samples were centrifuged at 1000 rpm for 5 minutes at room temperature. The supernatant was then collected and analyzed for LDH release using a commercial LDH cytotoxicity assay kit, following the manufacturer’s protocol.

1.4 Calcein/PI Staining Assay

Cell viability and death were assessed using Calcein/PI dual staining. HK-2 cells were seeded into 12-well plates and incubated at 37°C with 5% CO₂ for 24 hours to ensure proper cell attachment. Cells were then treated according to the experimental design. After treatment, the medium was removed, and cells were gently washed once with phosphate-buffered saline (PBS) to eliminate residual culture medium. The working solution was prepared following the manufacturer’s instructions for the Calcein/PI Cell Viability and Cytotoxicity Assay Kit. Added 500 μL of the working solution to each well and incubated at 37°C in the dark for 30 minutes. Fluorescence staining was then visualized using an inverted fluorescence microscope.

1.5 Dihydroethidium (DHE) staining

To assess reactive oxygen species (ROS) levels in renal tissues, DHE staining was performed on frozen kidney sections. Sections were incubated with DHE (10 μM) at 37°C for 30 minutes in the dark to visualize superoxide generation. Images were captured using the co-focal microscopy (Lecia).

1.6 Nanoparticle characterization

1.6.1 Dynamic light scattering (DLS) and zeta potential

Hydrodynamic diameter, polydispersity index (PDI), and zeta potential were measured by dynamic light scattering using a Zetasizer Nano ZS (Malvern Instruments, UK). Samples were diluted in deionized water to an appropriate concentration before measurement. Each value was obtained from triplicate measurements.

1.6.2 Encapsulation efficiency (EE) and drug loading (DL)

Encapsulation efficiency and drug loading were determined by quantifying EGCG content. Briefly, free EGCG in the supernatant after ultracentrifugation was measured by UV–Vis spectrophotometry at 210 nm. A standard calibration curve was generated by plotting OD values against known EGCG concentrations (0–50 μg/mL), which showed a good linear relationship (R² > 0.99). EE and DL were calculated using the following formulas: EE (%) = W_loaded_/W_total_ * 100; DL (%) = W_loaded_/W_NP_ * 100 (W_loaded_ is the weight of encapsulated EGCG, W_total_ is the total EGCG added, and W_NP_ is the weight of lyophilized nanoparticles).

1.6.3 In vitro release study.

The release profile of EGCG from TP-EGCG was evaluated using a dialysis method. Nanoparticles were dispersed in PBS (pH 7.4) or acetate buffer (pH 6.5) and placed in dialysis bags (MWCO 10 kDa), then immersed in 20 mL of release medium containing 0.5% Tween 80 to maintain sink conditions. Samples were incubated at 37 °C under gentle shaking, and aliquots were collected at predetermined time points up to 14 days, with equal volumes of fresh medium replenished. EGCG concentration was determined by UV–Vis spectrophotometry at 210 nm using the standard calibration curve.

1.7 Pizzolato’s staining

As described in a previous study[2], paraffin-embedded kidney sections were deparaffinized in xylene and rehydrated through a graded ethanol series to distilled water. The sections were then incubated in freshly prepared Pizzolato’s staining solution, composed of equal volumes of 5% silver nitrate and 30% hydrogen peroxide, and exposed to ultraviolet light for 30 minutes. After thorough rinsing with distilled water, the sections were counterstained with nuclear fast red solution for 5 minutes, followed by conventional dehydration, clearing, and mounting.

**Reference:**

1. Burgess S, Small DS, Thompson SG. A review of instrumental variable estimators for Mendelian randomization. Stat Methods Med Res. 2017; 26: 2333–55.

2. Si Y, Liu L, Cheng J, et al. Oral Hydrogen-Rich Water Alleviates Oxalate-Induced Kidney Injury by Suppressing Oxidative Stress, Inflammation, and Fibrosis. Front Med. 2021; 8: 713536.

**Supplementary Tables**

Table S1. The characteristics of clinical samples.

Table S2. The detail information of antibodies used in Western Blotting.

Table S3. The primer sequence for qRT-PCR.

Table S4. List of differentially expressed genes.

Table S5. Details of plasmid vectors and siRNA sequences.

Table S6. Baseline characteristics of participants.

Table S7. Details of IVs utilized in the MR analysis of tea consumption and kidney stones.

Table S8. Pleiotropy and heterogeneity analysis of tea consumption with kidney stones.

**Supplementary Figures**


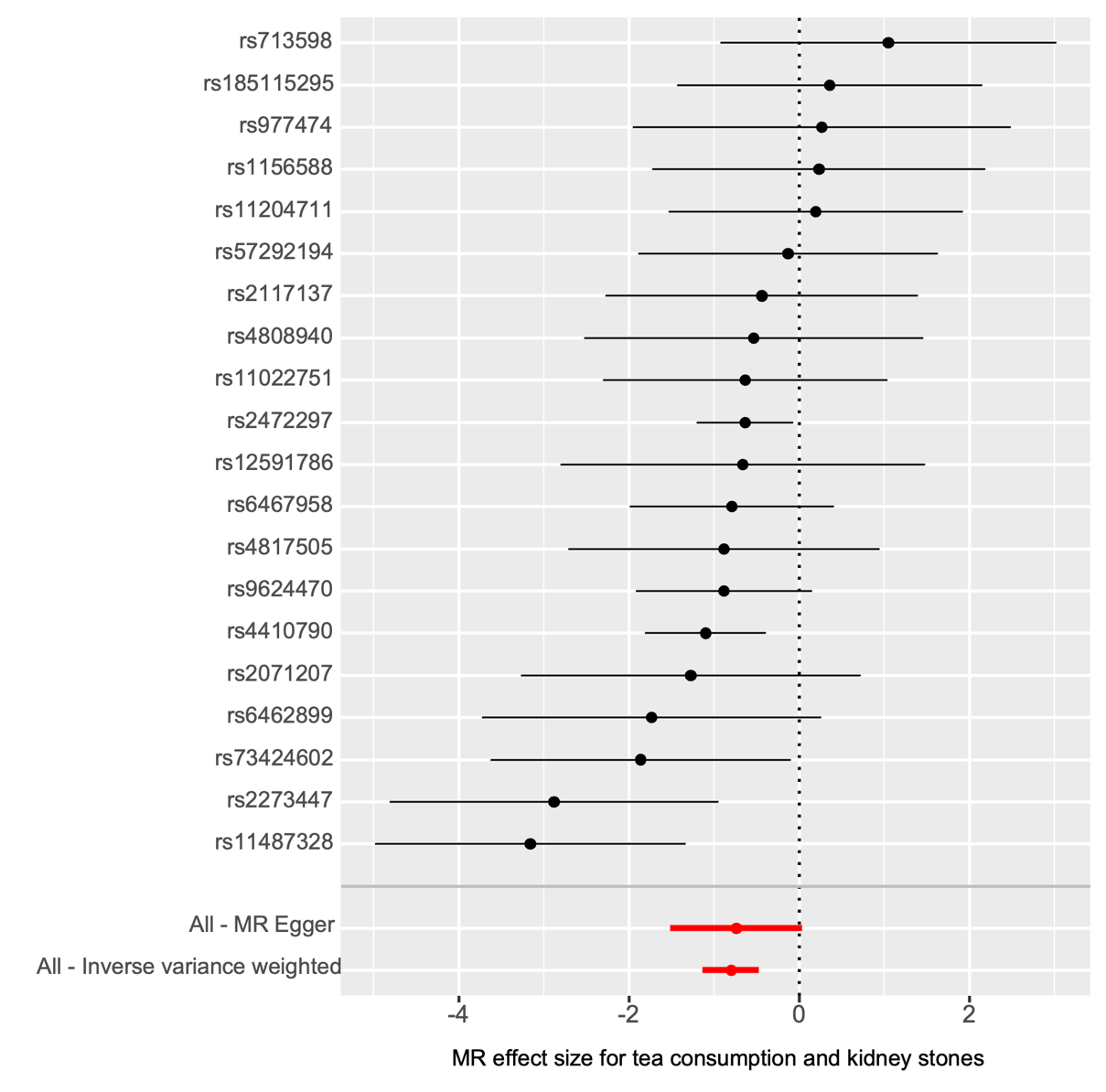


**Figure S1. Leave-one-out analysis for the relationship between tea consumption and kidney stones.**


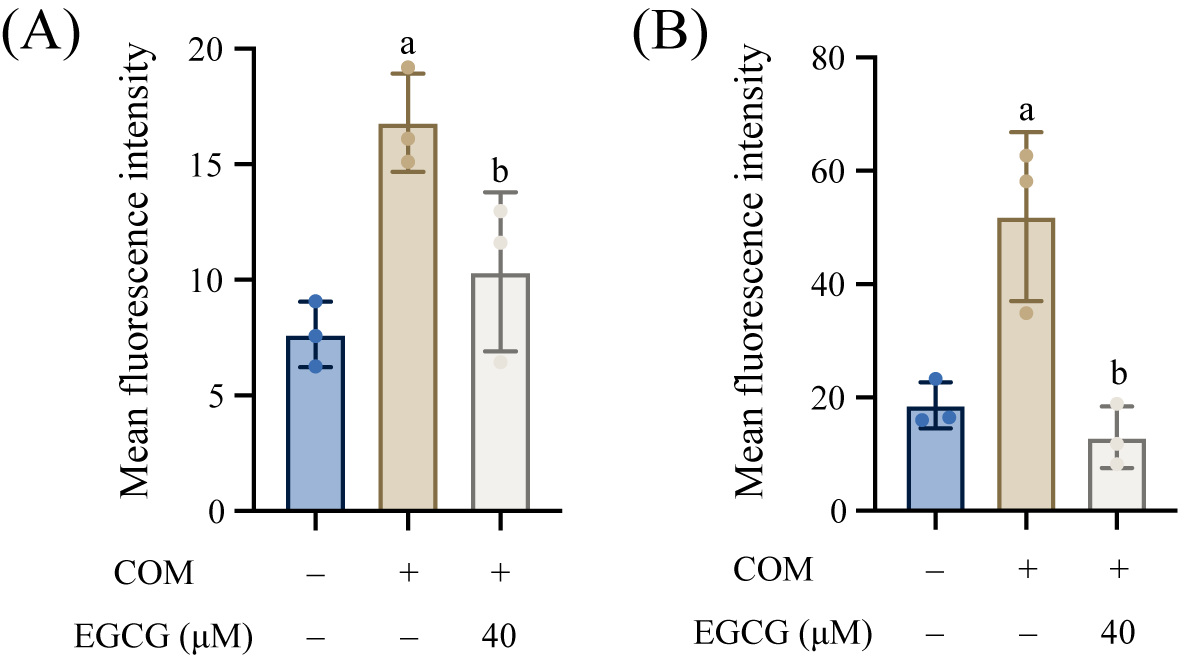


**Figure S2. Semi-quantitative analysis of immunofluorescence for the stone-related proteins CD44 (A) and OPN (B).** n = 3; One-way ANOVA followed by Tukey’s post hoc test was performed for multiple-group comparisons; a: *p* < 0.05 vs. the Control group; b: *p* < 0.05 vs. the COM group.


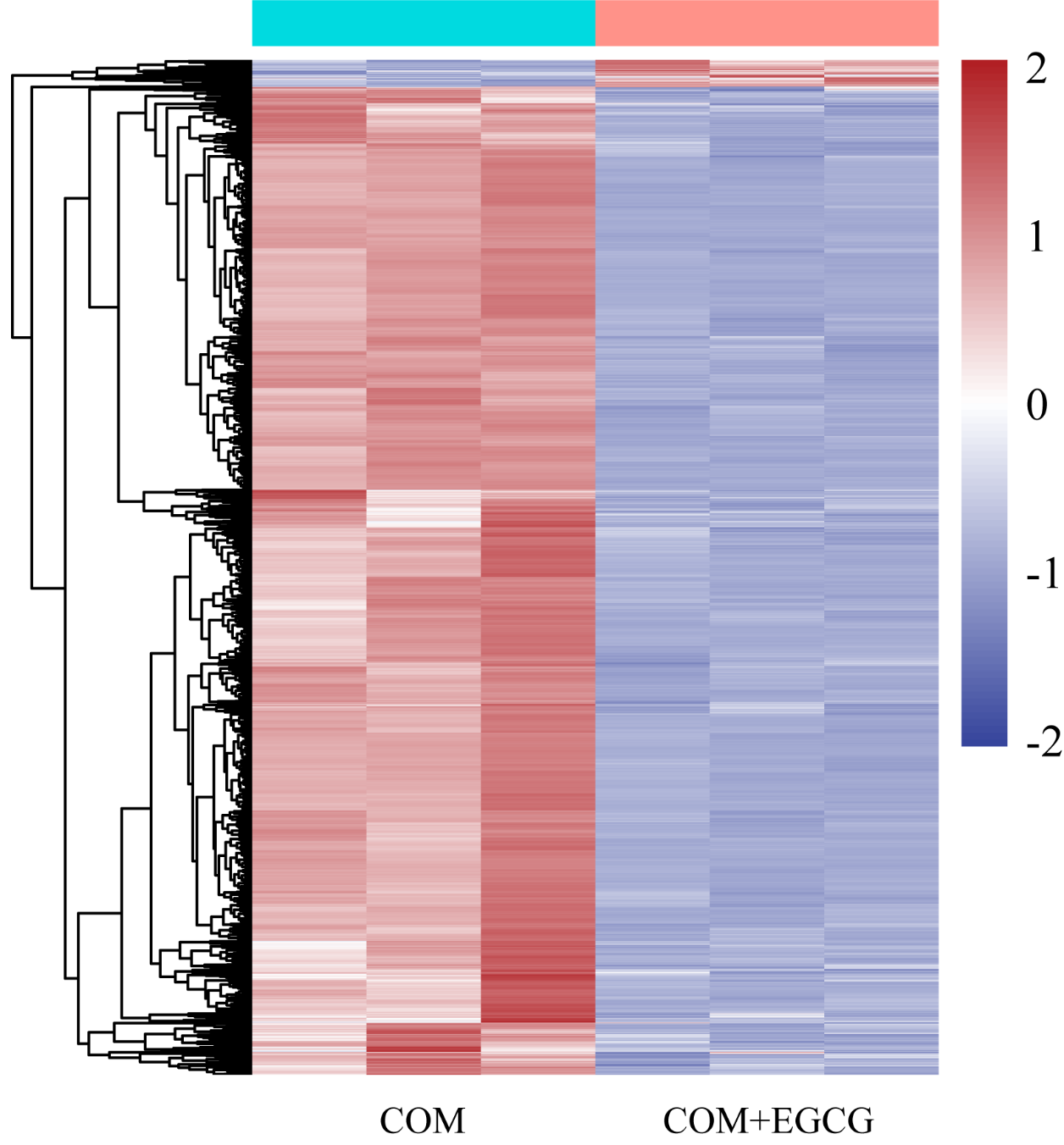


**Figure S3. Heatmap of Differentially Expressed Genes.**


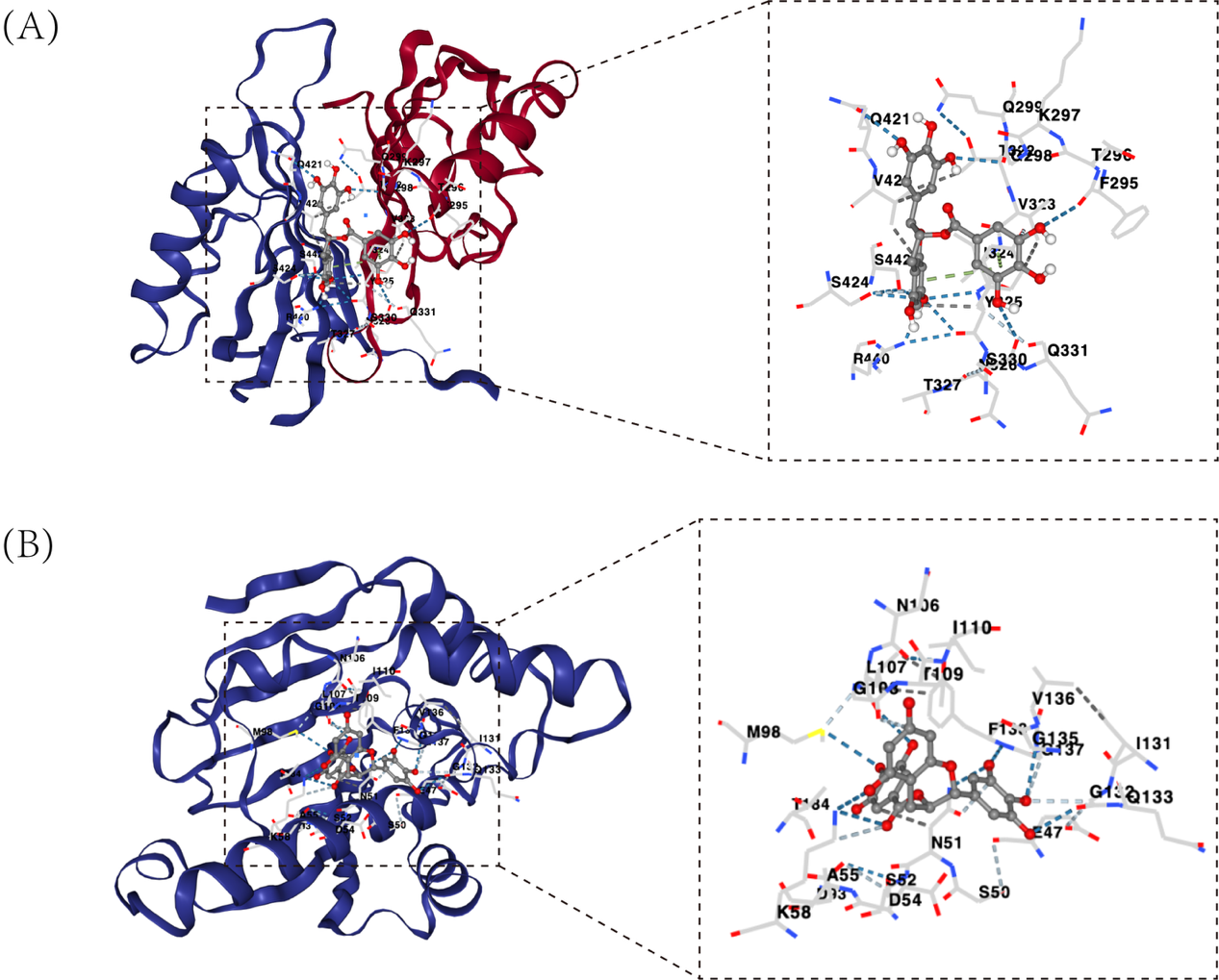


**Figure S4. Molecular docking results of EGCG with HIF1A and HSP90-α.** (A) Docking model of EGCG with HIF1A (affinity = -7.5 kcal/mol). (B) Docking model of EGCG with HSP90-α (affinity = -7.7 kcal/mol).


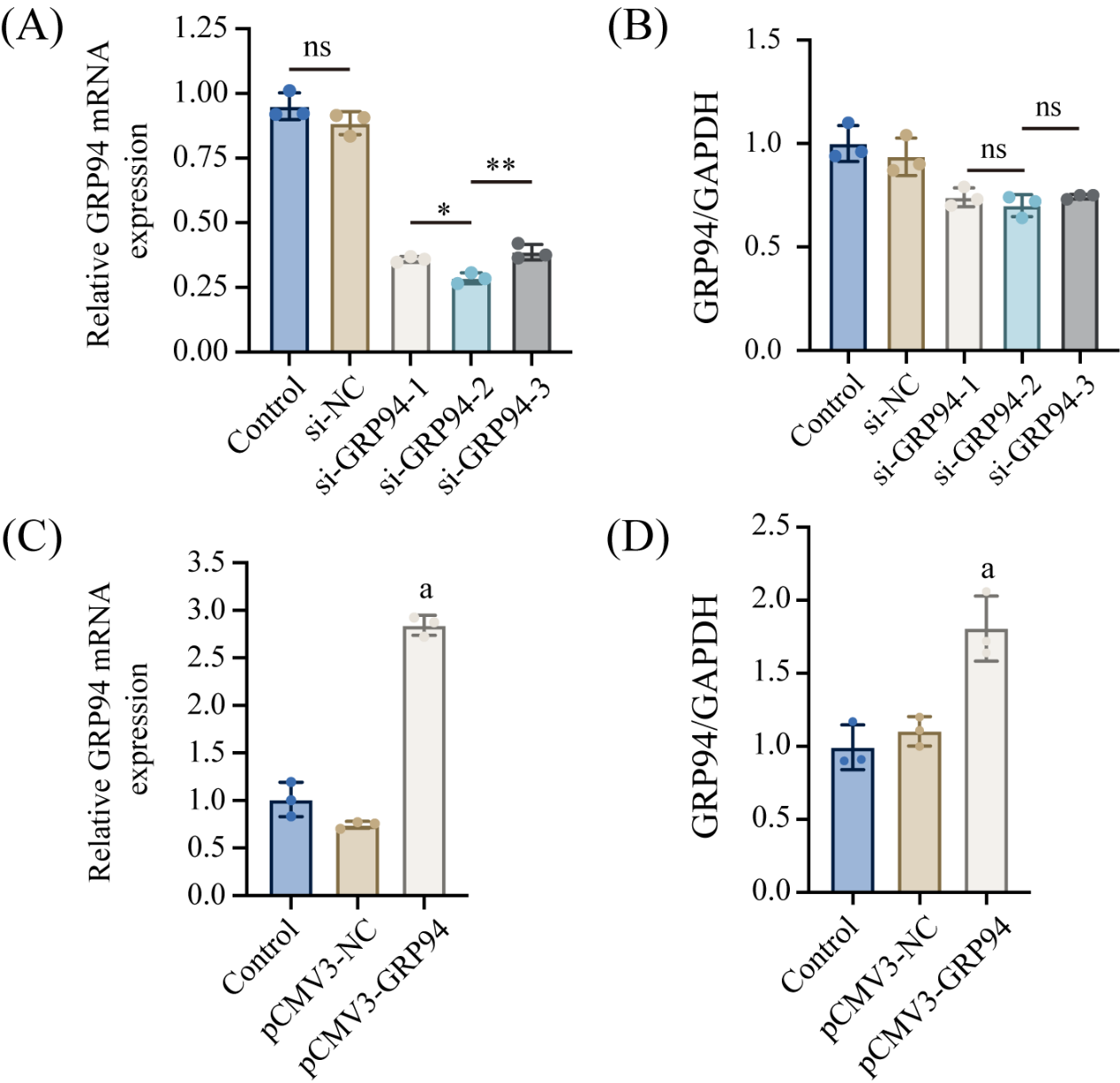


**Figure S5. Efficiency of GRP94 Knockdown or Overexpression.** (A) qRT-PCR analysis of GRP94 knockdown; (B) Semi-quantitative analysis of GRP94 knockdown by WB; (C) qRT-PCR analysis of GRP94 overexpression; (D) Semi-quantitative analysis of GRP94 overexpression by WB. n = 3; One-way ANOVA followed by Tukey’s post hoc test was performed for multiple-group comparisons; ns, not significant; *, *p* < 0.05; **, *p* < 0.01; a, *p* < 0.05 vs. Control group.


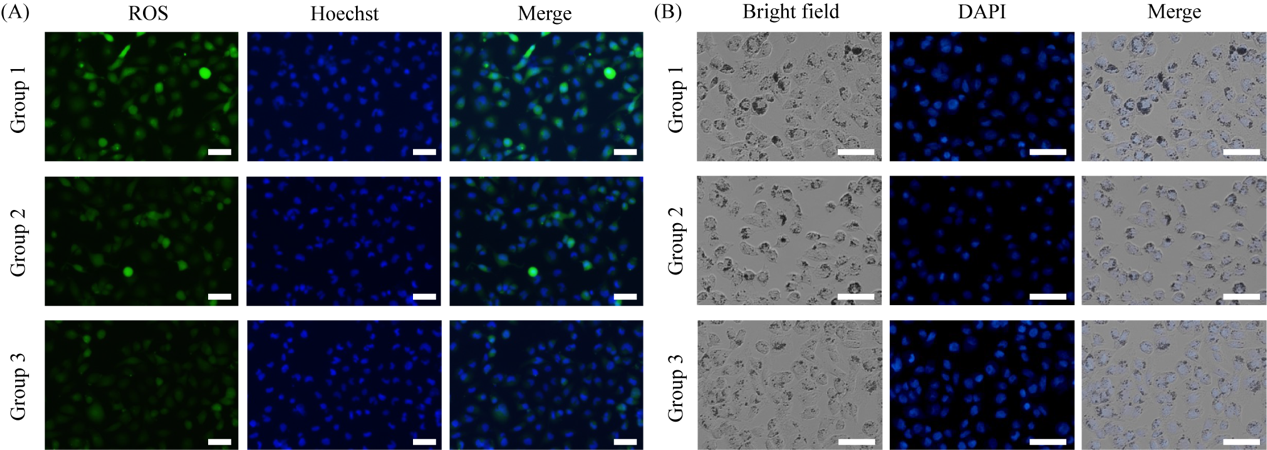


**Figure S6. Effects of GRP94 knockdown and PI3K inhibition on ROS production (A) and crystal adhesion (B) in COM-stimulated HK-2 cells treated with EGCG.** Group 1, COM + EGCG; Group 2, COM + EGCG + si-GRP94; Group 3, COM + EGCG + si-GRP94 + LY294002. Scale bar = 50 μm.


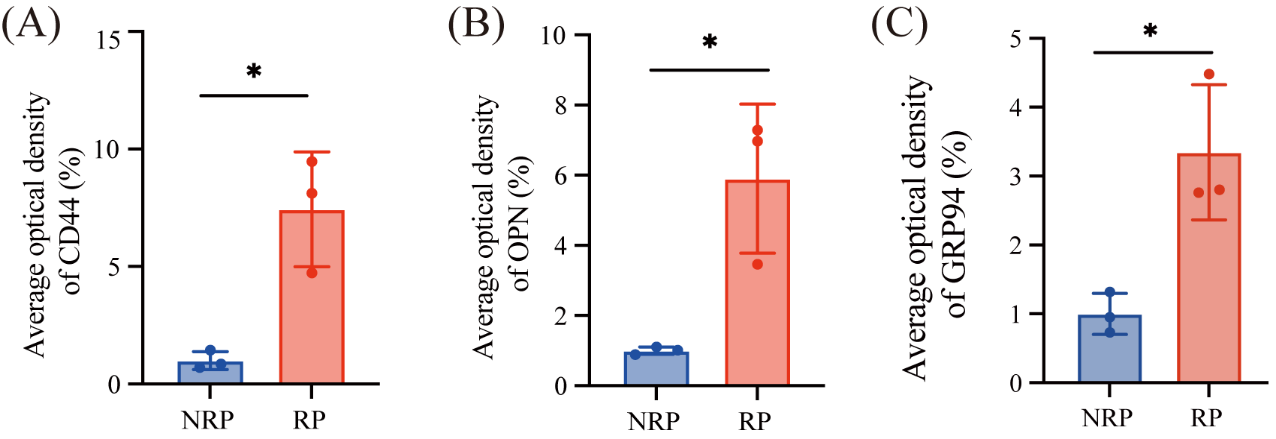


**Figure S7. Semi-quantitative analysis of Immunohistochemistry in NRP and RP Tissues (n = 3).** (A) CD44; (B) OPN; (C) GRP94. Student’s t test was performed for two-sample comparisons. * *p* < 0.05


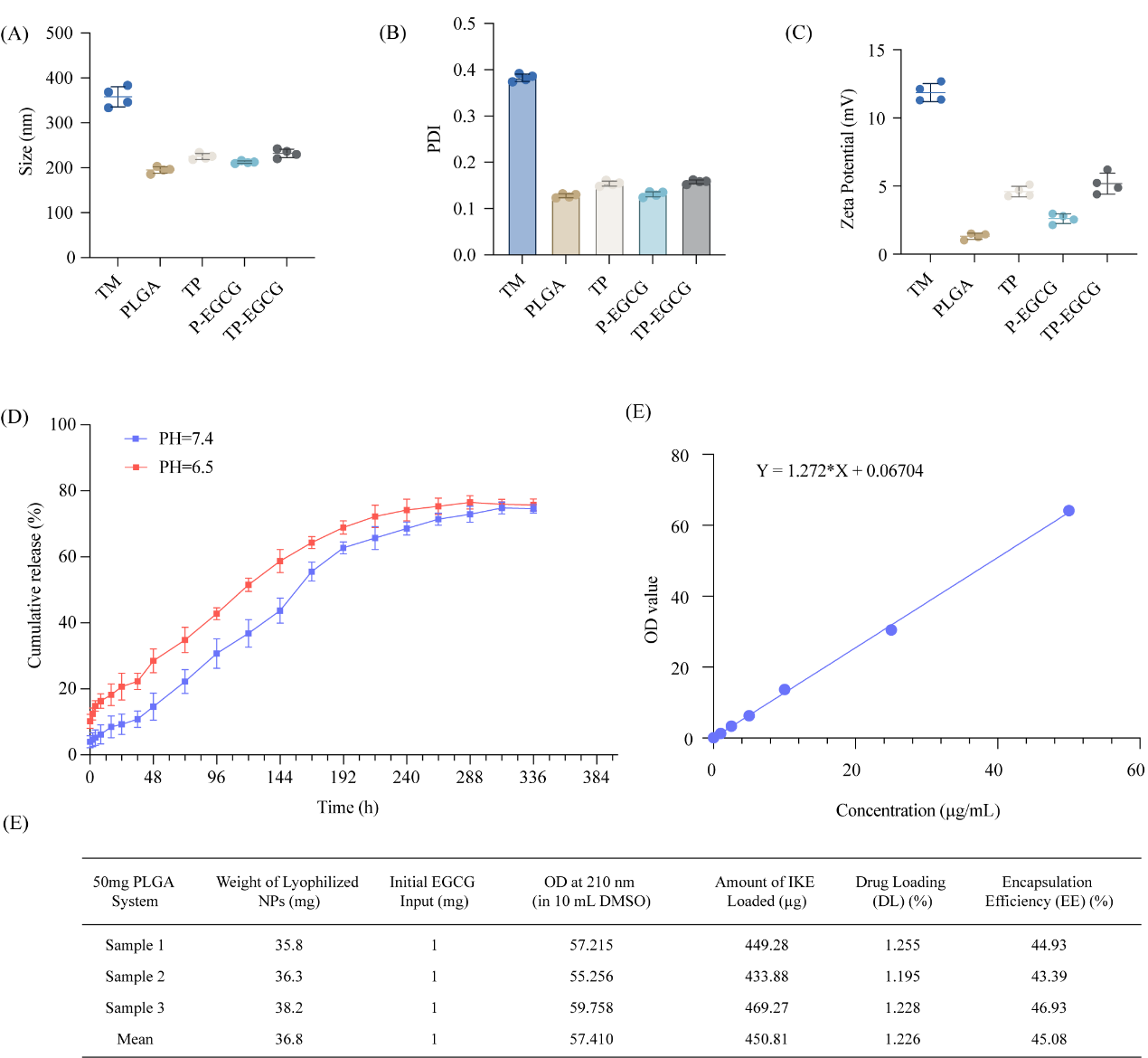


**Figure S8. Physicochemical characterization of TP-EGCG nanoparticles.** (A) Particle size distribution of different nanoparticle formulations (TM, PLGA, TP, P-EGCG, and TP-EGCG) measured by dynamic light scattering (DLS). (B) Polydispersity index (PDI) of the above formulations. (C) Zeta potential analysis indicating surface charge differences between formulations. (D) In vitro cumulative release profile of EGCG from TP-EGCG at pH 7.4 and pH 6.5. (E) Calibration curve of EGCG concentration versus OD value for quantification. (F) Encapsulation efficiency (EE%) and drug loading (DL%) of TP-EGCG determined from three independent batches.


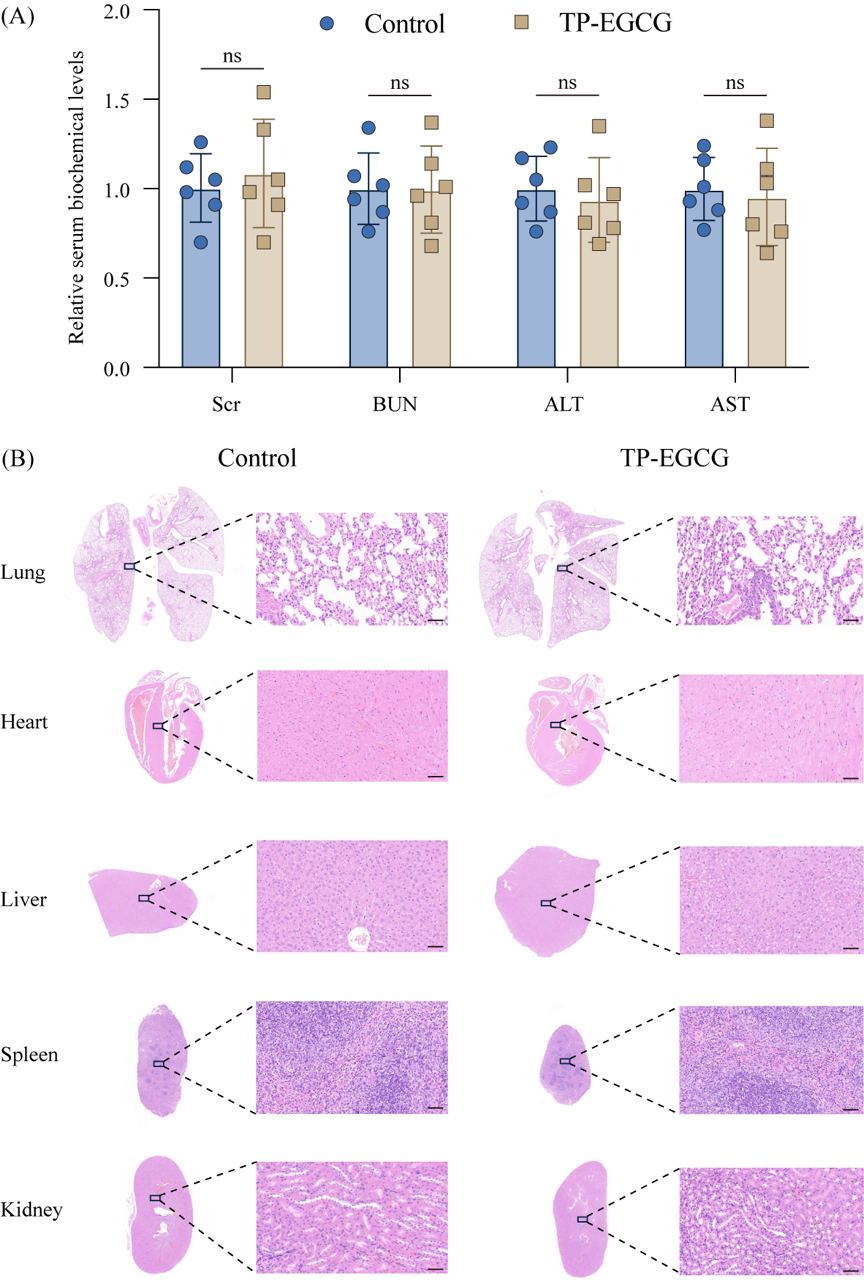


**Figure S9. Evaluation of the systemic safety of TP-EGCG *in vivo*.** (A) Serum biochemical parameters including serum creatinine (Scr), blood urea nitrogen (BUN), alanine aminotransferase (ALT), and aspartate aminotransferase (AST) were measured in mice after TP-EGCG treatment. Data are presented as mean ± SD; Student’s t test was performed for two-sample comparisons. ns: *p* > 0.05. (B) H&E staining results of lung, heart, liver, spleen, and kidney tissues from control and TP-EGCG intervention groups. Scale bar: 50 μm.


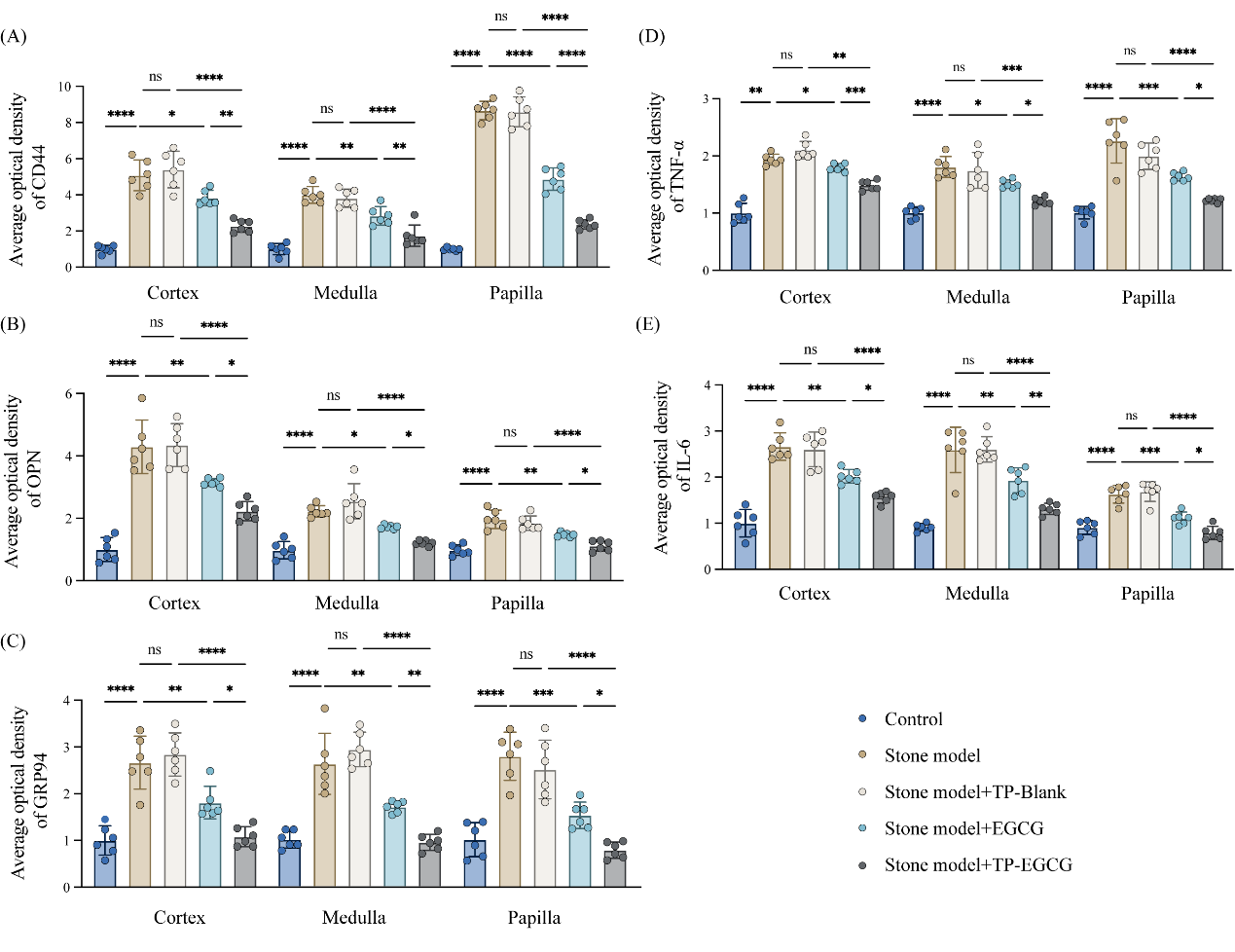


**Figure S10. Semiquantitative analysis of CD44 (A), OPN (B), GRP94 (C), TNF-α (D) and IL-6 (E) immunohistochemical staining in mouse renal tissues across experimental groups (Control, Stone model, Stone model + TP-Blank, Stone model + EGCG, and Stone model + TP-EGCG), n = 6.** One-way ANOVA followed by Tukey’s post hoc test was performed for multiple-group comparisons. ns, *p* > 0.05; *, *p* < 0.05; **, *p* < 0.01; ***, *p* < 0.001; ****, *p* < 0.0001.


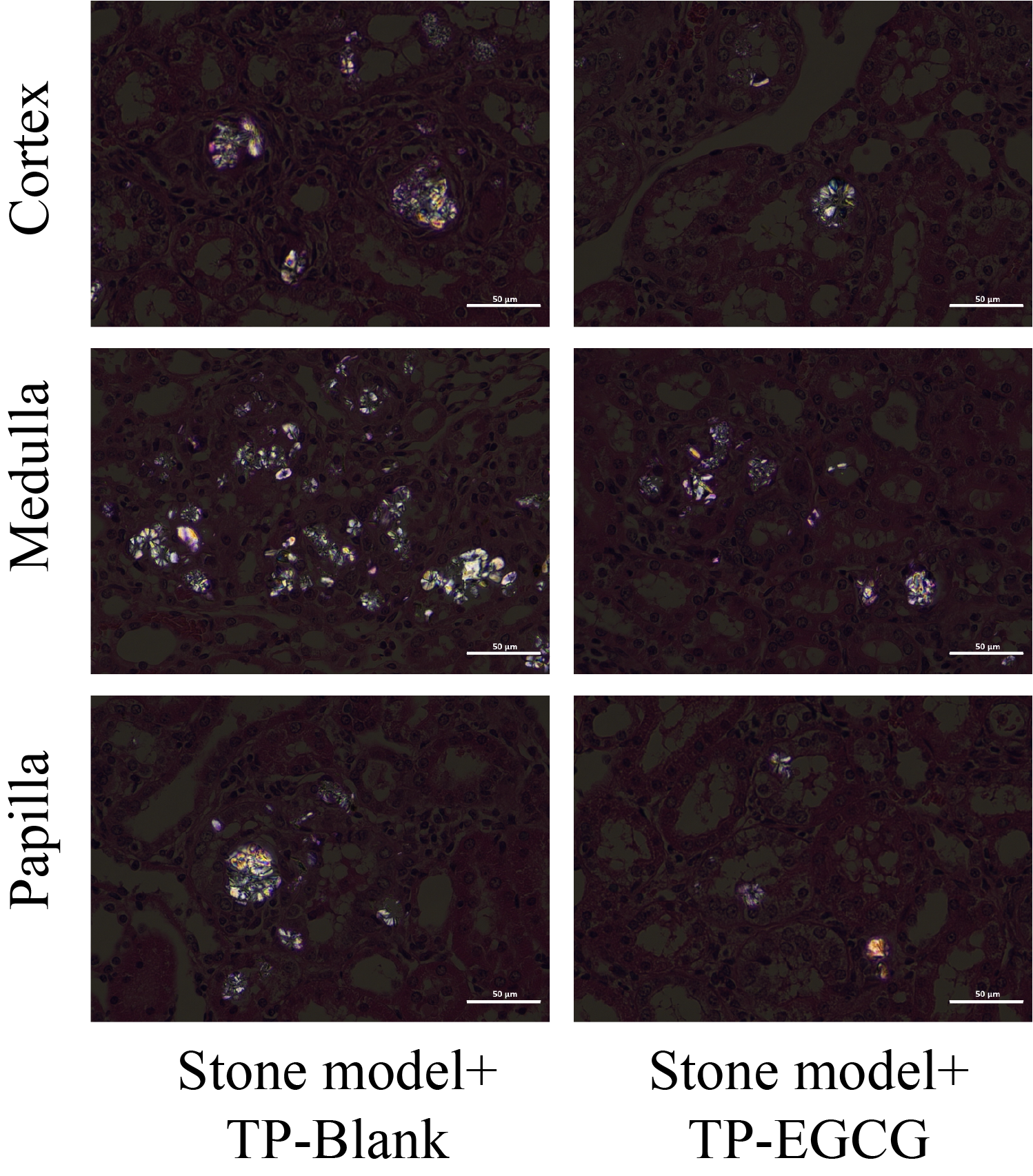


**Figure S11. CaOx crystal deposition was observed by polarized light microscopy. Scale bar: 50 μm.**
